# Coal dust exposure triggers heterogeneity of transcriptional profiles in mouse pneumoconiosis and Vitamin D remedies

**DOI:** 10.1186/s12989-022-00449-y

**Published:** 2022-01-20

**Authors:** Min Mu, Bing Li, Yuanjie Zou, Wenyang Wang, Hangbing Cao, Yajun Zhang, Qixian Sun, Haoming Chen, Deyong Ge, Huihui Tao, Dong Hu, Liang Yuan, Xinrong Tao, Jianhua Wang

**Affiliations:** 1 Key Laboratory of Industrial Dust Control and Occupational Health of the Ministry of Education, Anhui University of Science and Technology, Huainan, China; 2 Key Laboratory of Industrial Dust Deep Reduction and Occupational Health and Safety of Anhui Higher Education Institutes, Anhui University of Science and Technology, Huainan, China; 3 School of Medicine, Department of Medical Frontier Experimental Center, Anhui University of Science and Technology, 168 Taifeng Road, Huainan, China; 4 Anhui Province Engineering Laboratory of Occupational Health and Safety, Anhui University of Science and Technology, Huainan, China; 5 Cancer Institute, Fudan University Shanghai Cancer Center, Fudan University, Shanghai, China

**Keywords:** Single-cell RNA sequencing, Alveolar regeneration, Coal dust pulmonary disease, Epithelial cells, Macrophage subset phenotype activation, Pulmonary toxicity

## Abstract

**Background:**

Coal dust particles (CDP), an inevitable by-product of coal mining for the environment, mainly causes coal workers’ pneumoconiosis (CWP). Long-term exposure to coal dust leads to a complex alternation of biological processes during regeneration and repair in the healing lung. However, the cellular and complete molecular changes associated with pulmonary homeostasis caused by respiratory coal dust particles remain unclear.

**Methods:**

This study mainly investigated the pulmonary toxicity of respirable-sized CDP in mice using unbiased single-cell RNA sequencing. CDP (< 5 μm) collected from the coal mine was analyzed by Scanning Electron Microscope (SEM) and Mass Spectrometer. In addition, western blotting, Elisa, QPCR was used to detect gene expression at mRNA or protein levels. Pathological analysis including HE staining, Masson staining, immunohistochemistry, and immunofluorescence staining were performed to characterize the structure and functional alternation in the pneumoconiosis mouse and verify the reliability of single-cell sequencing results.

**Results:**

SEM image and Mass Spectrometer analysis showed that coal dust particles generated during coal mine production have been crushed and screened with a diameter of less than 5 µm and contained less than 10% silica. Alveolar structure and pulmonary microenvironment were destroyed, inflammatory and death (apoptosis, autophagy, and necrosis) pathways were activated, leading to pneumoconiosis in post 9 months coal dust stimulation. A distinct abnormally increased alveolar type 2 epithelial cell (AT2) were classified with a highly active state but reduced the antimicrobial-related protein expression of LYZ and Chia1 after CDP exposure. Beclin1, LC3B, LAMP2, TGF-ß, and MLPH were up-regulated induced by CDP, promoting autophagy and pulmonary fibrosis. A new subset of macrophages with M2-type polarization double expressed MLPH + /CD206 + was found in mice having pneumoconiosis but markedly decreased after the Vitamin D treatment. Activated MLPH + /CD206 + M2 macrophages secreted TGF-β1 and are sensitive to Vitamin D treatment.

**Conclusions:**

This is the first study to reconstruct the pathologic progression and transcriptome pattern of coal pneumoconiosis in mice. Coal dust had obvious toxic effects on lung epithelial cells and macrophages and eventually induced pulmonary fibrosis. CDP-induced M2-type macrophages could be inhibited by VD, which may be related to the alleviation of the pulmonary fibrosis process.

**Supplementary Information:**

The online version contains supplementary material available at 10.1186/s12989-022-00449-y.

## Introduction

Coal remains the primary source of electricity (60%) and iron production (40%) globally [1]. Coal dust hazards in coal mining have been studied continuously by the WHO (World Health Organization) and governments of many countries. As of 2018, pneumoconiosis accounted for 90% of occupational diseases reported in China's occupational health status reports, with 200 million people still at risk of occupational health exposure [2]. The rates of pneumoconiosis in Australia have started rising [3]. Many studies have clearly shown that coal particles have cytotoxicity, pro-inflammatory, pro-fibrotic properties, and a complex progress mechanism [4–6]. The most extensive study on pneumoconiosis in the U.S. revealed that respirable dust from coal miners caused progressive massive fibrosis (PMF) even after dust exposure has ceased [7, 8]. Mine Safety and Health Administration (MSHA) issued new regulations [9] for respirable coal dust concentration limit (from 2.0 to 1.5 mg/ m^3^). However, the prevalence rate continued to increase, with some regions such as the central Appalachian area seeing a sixfold increase [10]. There is no effective treatment for coal workers' pneumoconiosis.

Continued exposure to high concentrations of coal dust may lead to an inability to remove accumulated dust particles, resulting in structural damage to the alveoli [11]. Alveolar epithelial cells are the primary functional unit for O_2_ exchange and the first point of contact for inhaled particles [11]. Successful alveolar epithelial repair and functional recovery require the proliferation and differentiation of alveolar type 2 stem/progenitor cells (AT2). During the regeneration process, alveolar epithelial cells have been reported to have dysplasia or developmental delays in repair [12], which is associated with many chronic lung diseases [13]. Commonly, inflammatory monocytes and pulmonary macrophages are the keys to lung repair and fibrosis regulators. Lung macrophages in healthy adults are divided into mononuclear mesenchymal macrophages and tissue-resident macrophages that develop from embryonic precursors. Abnormal tissue niches can lead to the development of abnormally differentiated tissue-resident macrophages [14]. Alveolar macrophages (AMs), the central target cells of coal dust during phagocytosis and clearance, are related to oxidative stress and the activation of the innate immune system [15]. Exogenous pathways regulate apoptosis and the release of fibrotic factors from AMs. TNF-α, IL 6, and IL 1β are recognized pro-inflammatory factors released by macrophages [16]. VD3 can significantly inhibit activation of the TGF-β signaling pathway and up-regulation of fibronectin and collagen expression [11, 12].

The diversity of macrophages differentiates in coal pneumoconiosis, and the long-term coal dust-induced alveolar inflammatory mechanisms are worth studying.

Single-cell RNA sequencing (scRNA-Seq) technology can identify cell type-specific markers and provide definitions and functions of cell types at the molecular level. For example, Kyler et al. created a complete molecular cell map of the adult lung using scRNA-Seq, which peaked in the genome-wide expression profile of the lung [17]. The entire life cycle of alveolar type 2 cells was revealed during the maturation of bipotential progenitor cells in two alveolar lineages [18]. Recently, it was found that mouse embryonic lung tissue has rich progenitor cells with different ecological niches during early development [19]. Interestingly, the activation of mTORC1 in the lung stroma resulted in female-like changes in stromal and epithelial cells and decreased lung function [20]. In addition, many aging-related lung markers, including increased cholesterol biosynthesis in type 2 lung cells, adipose fibroblasts, and changes in the relative frequency of airway epithelial cells, are discovered [21]. The molecular mechanism of interstitial lung disease, which is often associated with degenerative changes in lung function, has been elucidated by scRNA-Seq. However, the diversity of macrophages differentiates in coal pneumoconiosis, and the long-term coal dust-induced alveolar inflammatory mechanism remains unclear.

Macrophages show diversity and adaptability in cell differentiation, phenotype, and function in response to lung disease and maintaining homeostasis. The heterogeneity of macrophages in bleomycin-induced pulmonary fibrosis in mice suggests that CX3CR1^+^SiglecF^+^ transition macrophages are localized to the fibrosis niche and have a fibrotic role in vivo [22]. Two independent interstitial macrophages (IM) subsets: Lyve1^lo^MHCII^hi^CX3CR1^hi^ (Lyve1^lo^MHCII^hi^) and Lyve1^hi^MHCII^lo^CX3CR1^lo^ (Lyve1^hi^MHCII^lo^) mononuclear derived IMs, have unique gene expression profiles, phenotypes, functions, and localization [23]. Furthermore, based on a new mouse model of macrophage depletion (Slco2b1^flox/DTR^), the loss of Lyve1^hi^MHCII^lo^ IMs exacerbates experimental pulmonary fibrosis [23]. The crosstalk between alveolar macrophages and lung epithelial cells at the single-cell level can broaden our understanding of alveolar epithelial repair. Although bleomycin-induced IPF changes in cell types, including activated macrophages, fibroblasts, and proliferative epithelial cells, have been explored using scRNA-Seq. However, few studies related to coal dust pneumoconiosis have been reported [22, 24, 25], the Single-cell heterogeneity, the regulatory mechanism, and the Vitamin D treatment for coal dust pneumoconiosis have not been studied. Vitamin D deficiency occurs in 90% of cases of pulmonary fibrosis [26, 27], especially in patients with coal workers' pneumoconiosis who have lower Vitamin D levels than healthy controls [28, 29]. Poor Vitamin D status is associated with pulmonary fibrosis [30, 31], whereas Vitamin D protects against particles-caused lung injury through induction of autophagy in an Nrf2-dependent manner, and it reduces lung injury and promotes tissue repair [32].

Here, an in-depth analysis of mouse lung macrophages and epithelial cells in 9-month post-coal exposure mice was performed, revealing the heterogeneity between the two populations in response to coal dust exposure using scRNA-Seq. In addition, the death signal analysis demonstrated the central roles of apoptosis-related pathways in death reprogramming in coal mice pneumoconiosis (CMP). Finally, cellular metabolism and communication landscapes were established within the single-cell resolution to explore better the interactions between AMs and epithelial cells in coal workers’ pneumoconiosis (CWP). Our findings will enrich the understanding of the cellular and molecular differences between normal and CMP lung tissues.

## Material and methods

### Chemicals and reagents

We used Vitamin D_3_[1,25(OH)_2_D_3_; VD_3_] (Sigma, product code: 740551, CAS: 128723-16-0, purity: 96%), and Vitamin D_3_ (Macklin Biochemical, product code: C804669, CAS: 67-97-0, purity: 98%) for the in vitro and in vivo experiments, respectively.

### Coal dust preparation

**Fig. 1:**
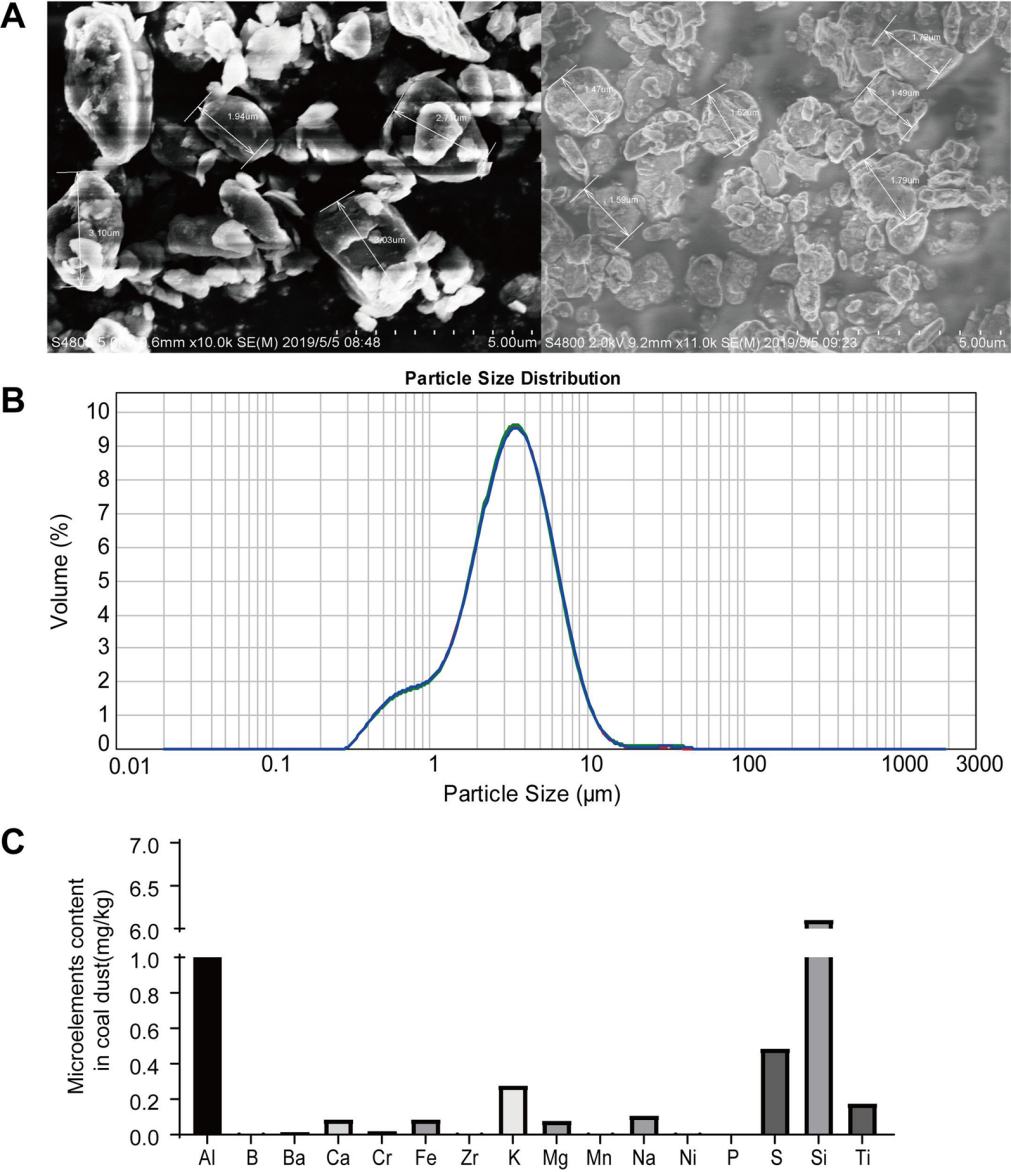
The chemical composition and particle size of coal dust are involved in this experiment. **A** The image of coal dust particles under scanning electron microscopy. All the particles are symmetrical, and the particle size distribution is narrow and has low silica content. **B** The distribution of coal dust particles is shown in the graph. **C** The chemical composition of coal dust particles was analyzed and presented in the table

We used the Gillian dust sampler to collect respirable coal dust in a coal mine in the Anhui Province, China. The distribution of particle size was detected by the Malvern laser particle size analyzer. Different particle size fractions were found (1 μm: 8.42%, 1–5 μm: 68.42%, and < 5 μm) and verified by SEM (Zeiss, EVO) (Fig. 1A, B). Particle size less than 5 microns accounted for 76.84% of the total particle number of dust samples. Each particle contained less than 10% silica revealed by ICP-MS (Agilent 7700) (Fig. 1C). Coal dust was weighed and autoclaved at 121 °C for 30 min before drying. Endotoxin detection was performed using the LAL test. The results showed that endotoxin was negative, < 0.01 EU/ML. Then, the coal dust was suspended in phosphate buffer saline (PBS) and sonicated for 15 min to ensure a uniform suspension before use.

### Animal husbandry

Male *C57BL/6* mice [license number SCXY (Su) 2011-0003] were bought from the Changzhou Cavion Experimental Animal Co. The mice were raised in cyclic light with free access to food and water. All animal experiments in the study followed the ARRIVE guidelines. All procedures were conducted following the guidelines described in the National Institutes of Health’s Guide for the Care and Use of Laboratory Animals (NIH Publication No. 8023, revised 1978) and were accepted by the Institutional Animal Care and Use Committee of the Anhui University of Science and Technology.

Mice (N = 42) aged 6–8 weeks were assigned randomly to three groups: control group (vehicle), coal group, and Vitamin D_3_ group, each consisting of 14 mice. After two weeks of adaptation mice weight were 22.55 ± 1.087 g. A time-escalating study of 3, 6, and 9 months was performed to evaluate the in vivo toxicity of coal dust. Coal dust (20 mg/ml/60 µl vs. saline) was administered intranasally into mouse lungs twice per week for four weeks with slight anesthetization [33]. Vitamin D3 was diluted by anhydrous ethanol and PBS and injected intraperitoneally in a volume of 0.1 mL three times per week at a dose of 400 IU/Kg until the experiment was completed.

### Hematoxylin and eosin (HE), Masson, and immunohistochemistry (IHC) staining

Mouse lung tissues for histological analysis were prepared through paraffin embedding or cryopreservation followed by formalin fixation. HE (Solarbio, Beijing, China) and Masson (Solarbio, Beijing, China) staining was performed on 5 μm-thick sections, and 10 μm-thick free-floating frozen sections were stained for double immunofluorescence staining. Roderick J's method [34] was used to conduct pathology score of lung injury and Ashcroft's method [35] was used to conduct fibrosis score. Deparaffinized sections were pretreated with heat-based antigen retrieval reagents and blocking reagents to prevent the non-specific binding of antibodies to tissue. Rabbit monoclonal anti-EMR1 (Proteintech, Wuhan, China), anti-MLPH (Proteintech, Wuhan, China), anti-LC3B (Abcam, Shanghai, China), and anti-CD206 (Proteintech, Wuhan, China) were used as the primary antibody. Horse anti-rabbit IgG Polymer Detection Kit, horse anti-mouse IgG Polymer Detection Kit (Vector, San Francisco, CA, USA) were applied before chromogenic substrate (Vector, San Francisco, CA, USA) was added. The nucleus was counterstained with hematoxylin. Alexa Fluor® 488 conjugate anti-mouse IgG (Cell Signaling Technology, Danvers, MA, USA), and Alexa Fluor®594 Anti-rabbit IgG (Abcam, Shanghai, China) were chosen for indirect double staining. DAPI (Beyotime, Shanghai, China) were counterstained for the nucleus. The sections were mounted with an antifade medium and observed under an Olympus FV3000 fluorescence microscope (Olympus, Tokyo, Japan), with all parameters (pinhole, contrast, and brightness) constant.

### Cell culture

Vitamin D3 can’t be converted into the active form 1,25(OH)2D3 by cultured cells. RAW264.7 cells were incubated with either 200 μg/mL coal dust, 1.2 × 10^–10^ mg/mL 1,25(OH)_2_VD_3_, or a mixture of 200 μg/mL coal dust and 1.2 × 10^–10^ mg/mL 1,25(OH)_2_VD_3,_ respectively, for 24 h. Subsequently, the cells were fixed with a 4% paraformaldehyde solution for 15 min. Anti-MLPH as the primary antibody, Alexa Fluor®594 conjugated goat anti-rabbit secondary antibody (Invitrogen, CA, USA) were used. DAPI dye stained for 10 min. Images were taken using a confocal laser scanning microscope (Olympus, Tokyo, Japan).

### Quantitative real-time PCR (qPCR) assay

Raw 264.7 cells were harvested, and total RNA was isolated using Trizol extraction (Thermo Fisher Scientific, Waltham, MA, USA). For cDNA synthesis, a 5 × All-In-One RT MasterMix kit (abm, Vancouver, Canada) was used. The real-time polymerase chain reaction was performed on a QuantStudio three Real-Time PCR Systems (Thermo Fisher Scientific, Waltham, MA, USA). For one amplification reaction, the reaction volume was kept at 10 μl by including 1 μl (10 ng) of cDNA, 5 μl of GoTaq® qPCR Master Mix (Promega, Madison, WI, USA), 0.4 μl of primer (10 μM), and DNase-free water. The primers are as follows: TGF-β, (FW)5-TGACGTCACTGGAGTTGTACGG-3, (RV)5´-GGTTCATGTCATGGATGGTGC-3´; GAPDH, (FW)5´-CCTCGTCCCGTAGACAAAATG-3´, (RV)5´-TGAGGTCAATGAAGGGGTCGT-3´. The relative abundance of transcripts of each gene was calculated according to the comparative ΔΔCT method.

### ELISA

Raw 264.7 cells were cultured with coal dust for 24 h, the supernatants were collected, and the concentrations of TGF-β1 were measured using ELISA kits (Catalog: RK00057; ABclonal, Wuhan, China) according to the manufacturer's protocol.

### Western blot assay

Raw 264.7 cells treated with coal dust (200 μg/mL) and 1.25(OH)_2_VD_3_ (1.2 × 10^–10^ mg/mL) were harvested, and cell lysis was obtained in RIPA buffer (Beyotime, Shanghai, China). Supernatants were collected, followed by centrifuging at a speed of 10,000 rpm for 10 min, and the protein concentration was determined using a BCA protein concentration determination kit (Beyotime, Shanghai, China). Next, 30 μg of the protein sample was loaded on an SDS-PAGE gel and electrophoresed. The electrophoresed proteins were transferred onto a polyvinylidene difluoride (PVDF) membrane (Thermo Fisher Scientific, Waltham, MA, USA). After blocking, MLPH antibody or GAPDH (Cell Signaling Technology, Danvers, MA, USA) was added. Chemiluminescence was then used to detect the proteins with an ECL developer (Thermo Fisher Scientific, Waltham, MA, USA). Finally, the protein expression levels were quantitated using ImageJ software.

### Quantification and statistical analysis

Statistical analyses were performed using the SPSS Statistics for Windows, version 11.0 (SPSS Inc., Chicago, IL, USA), and statistical significance was set at *p* < 0.05. The data are presented as means ± SEM. Statistical comparisons were performed using unpaired Student’s *t*-tests for two-tailed p values (*p < 0.05, **p < 0.01, and ***p < 0.001).

#### Tissue processing

Samples from the coal spots and adjacent normal *C57BL/6* mouse lung tissue from the same resection specimen were isolated and transported rapidly to the research facility. Each sample was subsequently minced on ice to less than 1-mm cubic pieces, followed by enzymatic digestion using DNase I (Worthington; 30 U mL 1), collagenase IV (Worthington; 195 U mL 1), collagenase I (Worthington; 10 U mL 1), and 30% FBS for 1 h at 37 °C, with manual shaking every 5 min. Then washing, cell resuscitation, lysis of red blood cells, filtration, cell count were as followed [36].

#### Advanced analytical methods for biological information

After Single-cell RNA sequencing [36] and Single-cell RNA-seq data preprocessing [37], we performed the following advanced bioinformatics analysis methods: sub-clusters analysis, differential gene expression analysis, Gene Ontology (GO), KEGG pathway analyses [38], Gene set variation analysis (GSVA) [39], Gene set enrichment analysis (GSEA) [40], Cell cycle analysis [41], Pseudotime analysis [42], Cell–cell communication analysis [43–45]. Additional details are provided in the Supplemental data.

## Results

### Successful establishment of coal mice pneumoconiosis by exposure to coal dust particles

**Fig. 2:**
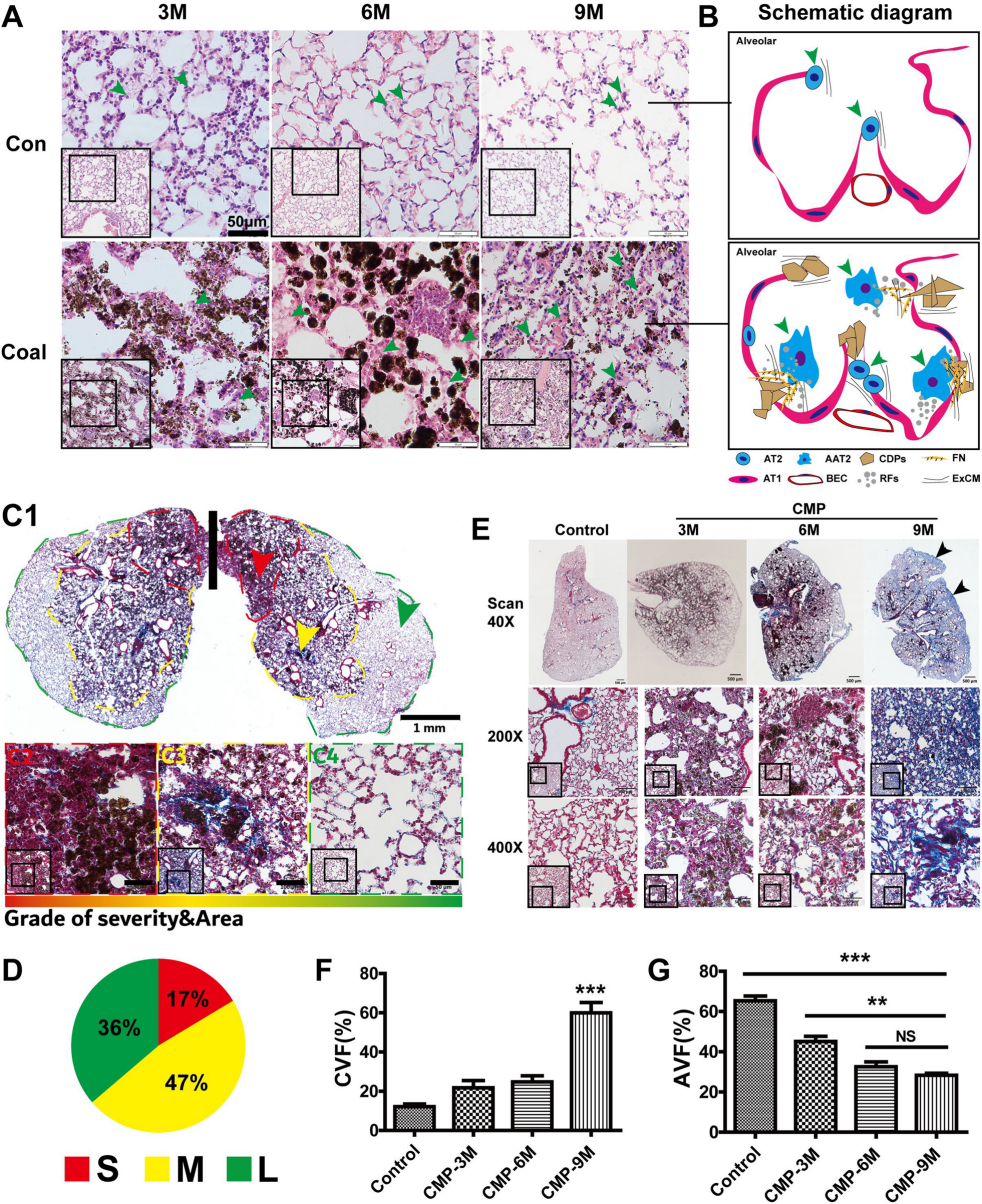
Dispersion of the inhaled coal dust into murine lungs and induction of lung fibrosis progression. The coal dust suspension was instilled via the nose, b.i.w., to accumulate 20 mg coal dust weight. Mice were examined at 3th, 6th and 9th months post-exposure. **A** Representative histological images (H&E) show the progress of pneumoconiosis tissue injury at 3, 6, and 9 months post-exposure. Green arrowheads pointed to the AT2. **B** Schematic diagram depicting lung injury and repair during coal pneumoconiosis at 9 months post-exposure (AT1: alveolar epithelial cell type 1, AT2: alveolar epithelial cell type 2, AAT2: Activated alveolar epithelial cell type 2, CDPs: coal dust particles, FN: fibronectin, RFs: repair related factor, BEC: blood endothelial cells, ExCM: extracellular matrix). **C1**–**C4** A representative sample of lung tissue showing a central-to-peripheral decline of lung interstitial cell density at 6 months, graded as severe (S), moderate (M), or light (L). **D** The lung section is divided into three regions, based on the density gradient of interstitial cells, representing severe, moderate, and mild damage. The proportion of the area of different lung interstitial cell density grades at the 6th month. **E** Representative histological images (Masson trichrome) showing pulmonary fibrosis progression in mice lungs at 3, 6, and 9 months after coal dust exposure (black arrows pointed to peripheral fibroblasts), 40× and 100× magnifications. **F** Collagenous volume fraction (CVF) at 3, 6, and 9 months, the area of collagen staining in lung tissue. **G** Alveolar volume fraction (AVF) at 3, 6, and 9 months, the area of the alveolar air cavity gradually shrinks. Both AVF and CVF were measured by free software Image J (NIH, http://rsbweb.nih.gov/ij/). The 40×, 200×, 400 × visual field were used and Semi-quantification under 200x (*p < 0.05, **p < 0.01, ***p < 0.001)

By exposing mice to coal dust twice-weekly for four weeks, we established a pathological phenotype of coal pneumoconiosis in these mice. Using this model, we studied the severity of coal dust lung damage at three, six, and nine months post-exposure, mainly reflected in the injury and repair of alveolar structures (Fig. 2A). In the third month, coal dust was embedded in the spaces between lung tissues, and cell proliferation resulted in alveolar stroma thickening. The coal dust particle has been phagocytosed by macrophages and aggregated into a ball, with lymphocytes proliferating into nodules in the sixth month. Finally, in the ninth month, the coal dust particles were again dispersed in the alveolar area, and the alveolar structure was destroyed and discontinuous. Roderick J. pathological grading of lung injury showed that the scores of coal dust exposed mice increased (***P < 0.001) significantly at three, six and nine months (Additional file 5: Fig. S5A). The significant increase in AT2 cells and the activation of repair functions resulted in the extracellular matrix deposition. Epithelial wounds repaired by infiltrating immune cells are shown in Fig. 2B, as illustrated by the diagram.

These results suggested that this technique was a reliable method for modeling CMP, as it produced an asymmetrical distribution of coal dust on both sides of the lungs. In addition, the inflammatory injuries in the lungs presented a trend of severe, moderate, and mild from the hilum to the lung margin (Fig. 2C1–C4). These three levels presented obvious boundaries (as delineated by the red, yellow, and green lines), and the proportions of affected areas were 17%, 47%, and 36%, respectively (Fig. 2D). The progression of pulmonary fibrosis in these mice at three, six, and nine months was demonstrated by Masson staining (Fig. 2E). Experimental coal pneumoconiosis in the mice had been latent for an extended period of six months before the formation of significant fibrosis. However, nine months after coal dust exposure, there was significant interstitial pulmonary fibrosis with a darker toluidine blue margin, suggesting an idiopathic centripetal fibrosis-like progression. Pulmonary fibrosis score performed by Ashcroft criteria for grading lung fibrosis showed that the scores of coal dust exposed mice increased (***P < 0.001) significantly at three, six and nine months (Additional file 5: Fig. S5B). The collagenous volume fraction (CVF), which reflects fibrosis progression, was quantitatively assessed, as was the alveolar volume fraction, an indicator for lung function in mice. There was a significant increase in CVF in nine months after coal exposure (*p* < 0.001) (Fig. 2F). With the increase of CVF in the development of fibrosis, the alveolar volume fraction (AVF) of the mice decreased (Fig. 2G), indicating a decline in respiratory function. Moreover, the Penh value was higher and the values of Mv, F, Av, EF50, PIF, PEF, Rpef were lower (*P < 0.05, **P < 0.01, ***P < 0.001) in the coal dust group compared with the control group (Additional file 5: Fig. S5D). This result indicated that the mice had increased respiratory resistance and decreased lung capacity at nine months after coal dust exposure. The mice weight decreased in the first month after coal dust exposure, but increased continually after coal dust ceased. However, the average weight of the coal dust group mice was lower than that of the control ones from eight to nine months (Additional file 5: Fig. S5C). At nine months, mice exposed to coal dust were subject to spontaneous death, a disease outcome associated with fibrosis progression.

### Classification of lung cells based on cell-type specific marker genes

**Fig. 3:**
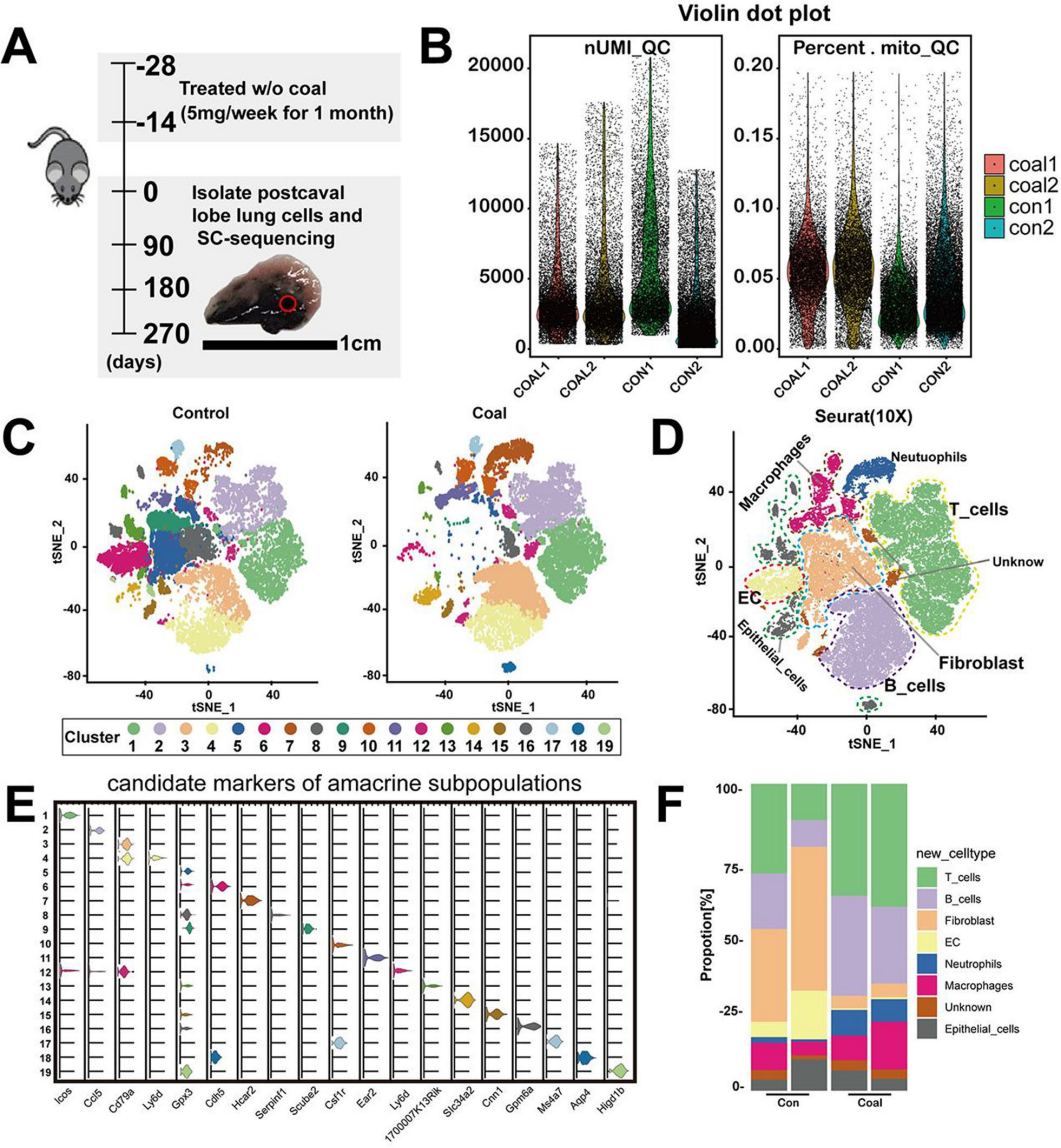
Single-cell landscape in murine lungs exposed to coal dust. **A** Schematic of the experimental workflow used to define the pulmonary cells of 9-month-exposed mice. **B** Quality control of unique cell molecular identifiers (UMI) and mitochondria. **C**, **D** tSNE plot and clustering of 42,252 cells from coal-exposed mice (n = 2) and vehicle control mice (n = 2), classified into 19 clusters and seven distinct cellular types. **E** Violin plot showed the feature of vital lineage-associated in the mouse lung clusters. **F** Average proportion of eight main types of lung cells in coal dust-exposed lungs and control lungs

According to the experimental flow chart (Fig. 3A), single-cell suspensions of 9-month-exposed murine pulmonary cells were prepared. We isolated and sequenced 50,156 cells from whole lung cell suspensions of four male mice, including two vehicle control mice and two coal-exposed mice. We first cataloged mouse lung cell types in an unbiased manner using droplet-based scRNA-Seq on the 10 × Genomics platform and performed analyses using a single-tube protocol with unique transcript counting through barcoding with UMIs. Then, the effects of UMI and mitochondrial gene content were examined (Fig. 3B). The clustering of cells was not affected by UMI or the mitochondrial gene content. Besides, genes positively correlated with mitochondria-encoded proteins were found associated with solute transport rather than cellular stress responses. After quality filtering, ~ 50 million unique transcripts were obtained from 42,252 cells, in which more than 1,303 genes could be detected. Of these, 24,094 cells (48%) originated from coal pneumoconiosis-infected lungs and 26,062 from control lungs (Additional file 8: Table S1). We performed dimensionality reduction with t-SNE subspace alignment, followed by an unsupervised clustering assay (Fig. 3C). To characterize the heterogeneity of coal exposed-pulmonary cells at a higher resolution, we focused on seven clusters from 12,855 cells of coal-exposed and control lungs (Fig. 3D). 19 distinct cell clusters consisting of as few as 1186 cells to 15,665 cells per cluster were classified. The representative markers identified 19 clusters, including epithelial cells (*Epcam, Krt18*), endothelial cells (*Pecam1, Cdh5*), fibroblast cells (*Dcn, Col1a1*), macrophages (*Cd114, Cd68, Csf1r*), monocytes (*CD14, CD300E*), neutrophils (*Arg2, Sorl1*), and lymphocytes (*CD79A, CD19, TRBC2*) (Fig. 3C, E). All the clusters included cells from both coal dust-treated and untreated lungs, except for cluster 19, which included cells mostly from coal dust-treated lungs (Additional file 1: Fig. S1A). Two superclusters were categorized and focused: one consisted of clusters 13, 14, 16, and 18 with higher Krt18 and EPCAM expression, and the other consisted of clusters 10, 11, and 17 with higher CD14 and CD68 expression (Additional file 1: Fig. S1B&C). Cluster 10 with the expression of CD14 and CD300E was recognized as proliferating cells. Clusters 11 and 17 expressed macrophage markers, such as CD14 and CD68. Cluster 18 expressed epithelial cell markers, such as ITGA1 and SCNN1G, and the highest level of Aqp4 (Fig. 3E). The cell-type composition distribution of each mouse was observed to differ substantially (Fig. 3F). As expected, immune cells, such as lymphocytes and macrophages, were the dominant cell type for coal pneumoconiosis.

### Intrinsic epithelial cell subpopulations underlying coal pneumoconiosis injury

**Fig. 4:**
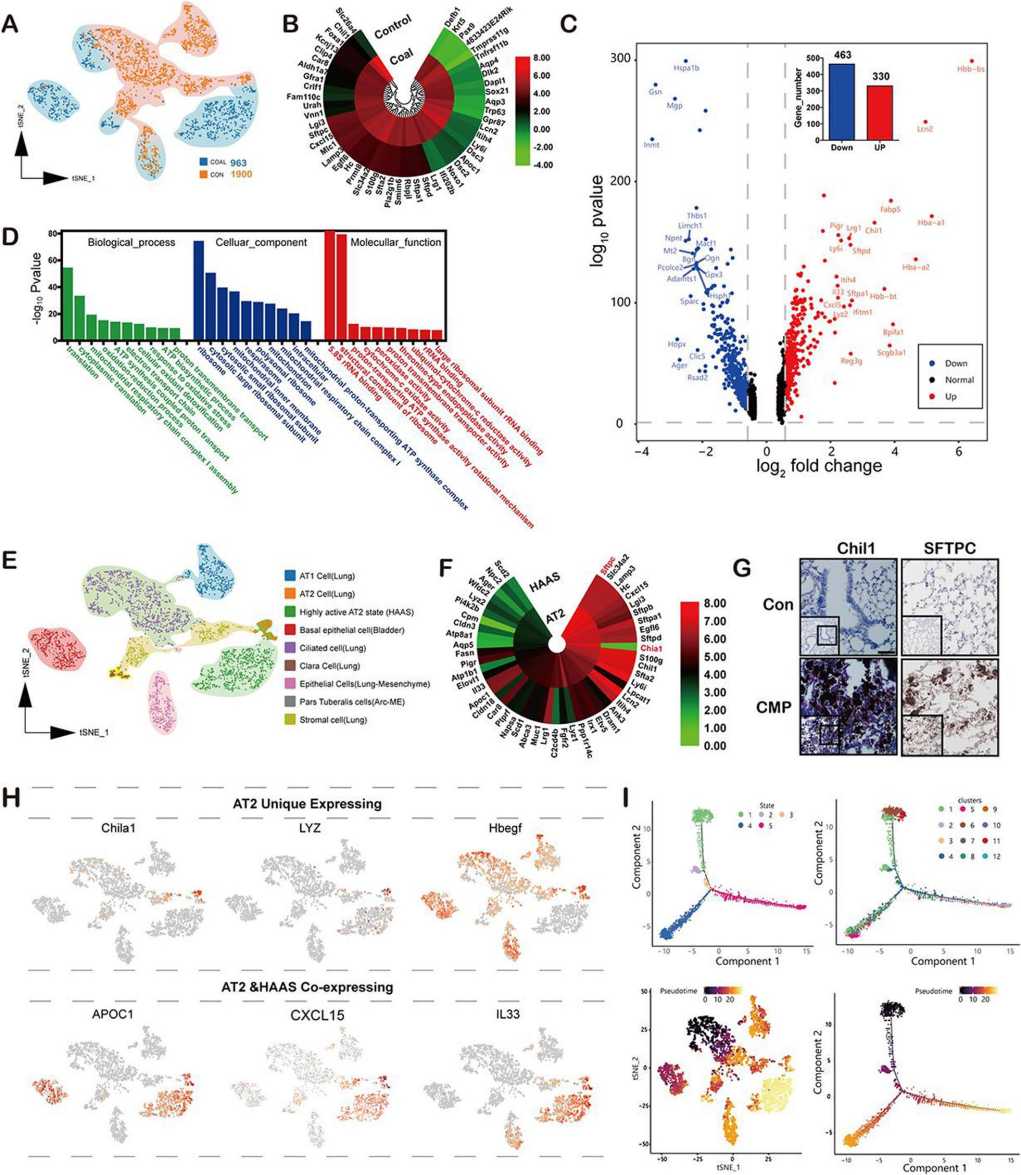
Longitudinal single-cell RNA-seq reveals the heterogeneity of epithelial subtypes and alveolar epithelial regeneration after long-term exposure to coal dust. **A** The tSNE plot shows the changes in the distribution of the subtypes of epithelial cells 9 months after coal dust-mediated lung injury. **B** Differentially expressed genes (DEGs) from scRNA-seq were identified, and the top 50 DEGs are shown. **C** Volcano plots highlight differentially expressed genes in the coal dust-exposed and control pulmonary cells. Red dots indicate gene expression upregulation in coal dust-exposed mice, while blue dots indicate downregulation after coal exposure. Labels were added to DWFb1, Krt5, and the top 20 most DEGs. **D** Gene ontology (GO) classification provides putative gene functions for pulmonary cells in coal-exposed mice. **E** Single-cell annotation and unsupervised migration clustering identified nine subtypes of epithelial cells. **F** Based on the lung epithelial cell analysis, the annular heat map shows the significant DEGs out of the top 50 DEGs. The single-cell sequencing data revealed that these top 50 genes were differentially expressed in alveolar dual potent progenitor cells and type 2 alveolar epithelial cells. **G** CHIL1 and SFTPC overexpression in-coal exposed lungs were identified by immunohistochemical staining that compared the coal-exposed lung tissues to the control lungs. **H** The proliferating epithelial subsets expressed APOC1, CXCL15, and IL33, covering the primary epithelial cells with CHILA1, LYZ, and HBEGF. Cell markers were used to identify clusters as represented in the tSNE plot. **I** Using pseudotime ordering analysis, we successfully constructed the pulmonary epithelial cell lineage as a differentiation trajectory. Each branch shows a single-cell state. The top left plot is marked with developmental time, and the right plot is marked with cell states

We detected 2,863 epithelial cells, including both epithelial and stromal cells. All the cells were re-analyzed, yielding 12 clusters. As expected, given the hypervascular nature of the lungs, the epithelial cells were less abundant (963 cells) in the coal pneumoconiosis group (Fig. 4A). Next, we attempted to identify marker genes for each cluster and assign them to known epithelial cell types. In many cases, the unbiased cluster identifier was a known cell-type-specific marker, such as surfactant protein C (SPC) for AT2 cells, Hopx for alveolar type I cells (AT1), and keratin 5 (KRT5) for basal cells (Fig. 4E). Notably, we identified many additional markers, followed by some of the known markers. One set of 48 Clara cells was found in coal pneumoconiosis samples (cluster 10; marker genes Scgb1a1 and MUC5b), as well as three sets of lymphatic epithelial cells (PDPN^+^); two mainly were coal pneumoconiosis-derived (clusters 4 and 5; AQP3^+^ and AQP1^+^) and one controlled lung-derived (cluster 3; SCNN1G). To identify differentially expressed genes (DEGs) that may play an essential role in the defense response to coal dust in murine lungs, the CWP group (Additional file 8: Table S2) that inhaled coal dust was compared to the vehicle control group. 793 DEGs were identified using the standard FDR of < 0.01 and fold-change of 1.5, of which 330 were upregulated and 463 were downregulated (Fig. 4C). The downregulated genes included those involved in the heat shock protein family (Hspa1b, Hsp90aa1, and Hspa1a), calcium-regulated actin-modulating protein (Gsn, Mgp, and LIMCH1), and intercellular adhesion (Thbs1 and Vcam). The distribution of DEGs is illustrated using a volcano plot (Fig. 3C), where the red color represents elevated expression and blue represents decreasing expression levels. To visualize the gene expression profiles of the top 20 genes, we generated a heatmap using a ring diagram (Fig. 4B). To further tell the function of DEGs, 793 DEGs from mice with or without coal dust exposure were subjected to GO annotation (Fig. 4D). The results demonstrated that 5269 GO terms were annotated, including 3711 biological processes (9543 unigenes), 618 cell components (5,164 unigenes), and 940 molecular functions (3993 unigenes). Most of the differentially expressed genes were mainly involved in the biological processes of cells. The DEGs in this study were mainly involved in cellular processes (GO: 0098869), molecular function (GO: GO: 0032981), ribosomal function (GO: 1990932 and GO: GO: 0006412), and ATP biosynthetic processes (GO: 0006754), all of which indicate that numerous physiological and biochemical alternation in the epithelial cells during coal dust inhalation into the lung, an important role in the coal dust defense response. According to the epithelial subtype identification results, we classified the epithelial cells into nine types. Compared with the control group, coal pneumoconiosis type II epithelial cells were more and had a significant difference in dimensionality reduction clustering, which was defined as a highly active AT2 state (HAAS) (Fig. 4E), and focused on the reprogramming of AT2 epithelial cells (cluster 2 and cluster 9). Next, a direct comparison of HAAS and normal AT2 epithelial cells was performed. We found that the antimicrobial-related protein expression of LYZ and Chia1 was missing in the AT2 epithelial cells of mice with coal pneumoconiosis (Fig. 4F). Remarkably, the total read counts were 7- to 8- fold higher in HAAS than in normal AT2 epithelial cell clusters, accounting for 48% of the total coal pneumoconiosis epithelial cells (Additional file 8: Table S3). Using immunohistochemical analysis of independent coal pneumoconiosis samples for CHIL1, SFTPC, and HBEGF markers of clusters 2 and 9, we confirmed the presence of these cells as separate cellular entities, which were enriched in coal pneumoconiosis tissues (Fig. 4G). The analysis of hallmark gene expression signatures highlighted that, while the two-coal pneumoconiosis epithelial cell clusters showed some differences, most changes were between the control lung and coal pneumoconiosis-derived AT2 epithelial cells (Fig. 4H). In short, these results indicate that coal pneumoconiosis epithelial cells are remodeled to increase the number of new AT2 epithelial cells, thus contributing to coal pneumoconiosis regeneration and the repair of coal dust-injured epithelial tissue. Finally, to map cell types, states, and transitions during the regeneration of dynamic biological processes in coal pneumoconiosis, we performed a pseudotime analysis of the epithelial cells using Monocle. The data suggested two diverging cell fates, starting at clusters 6, 8, and 9, progressing toward clusters 1, 5, and 7 at one end, and cluster 2 at the other, with cluster 4 being a transitioning state spreading along the axis (Fig. 4I).

### Coal dust induces murine AT2 cell apoptosis and reprogramming

**Fig. 5:**
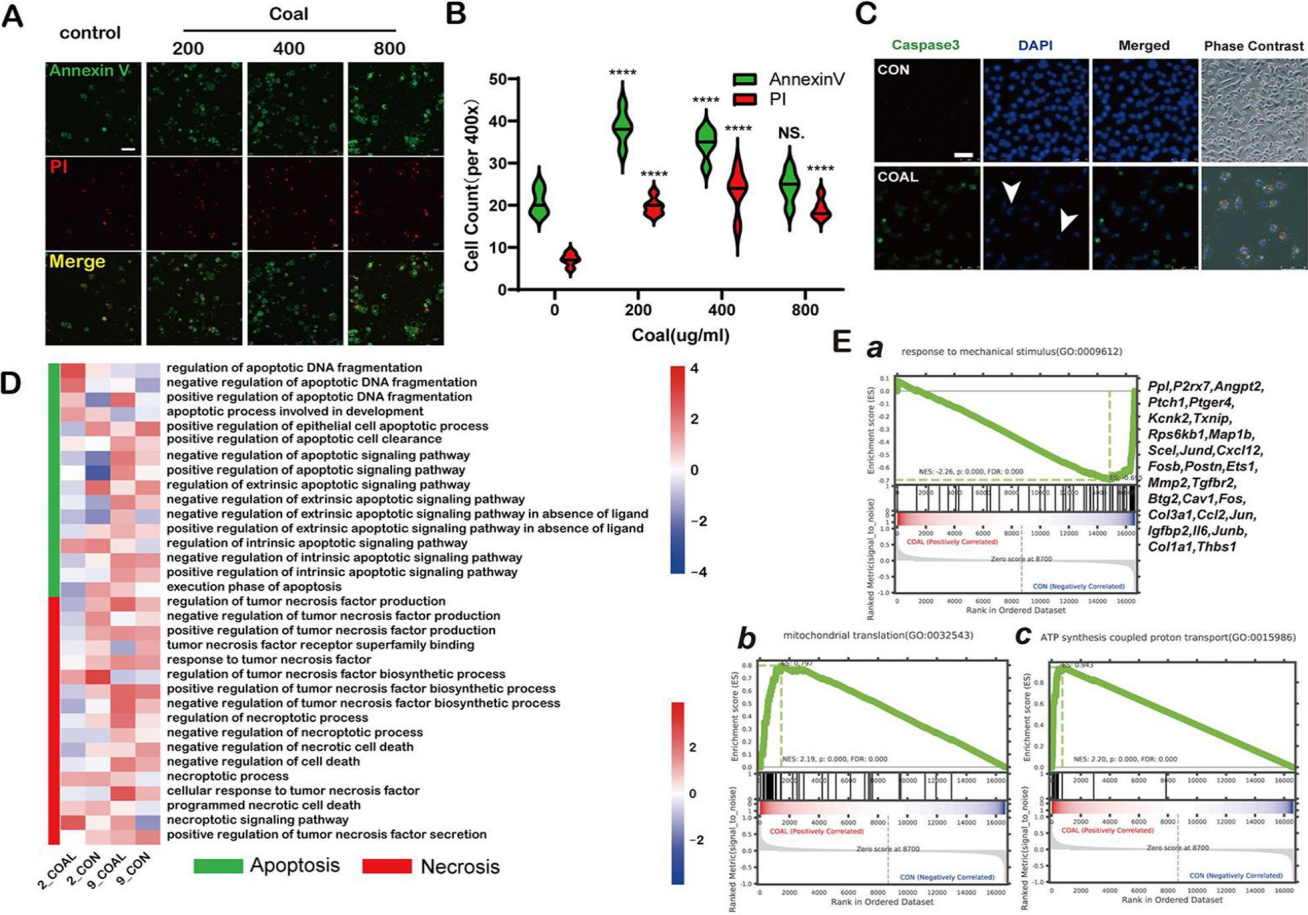
Coal dust exposure-induced pulmonary apoptosis and necrosis. **A** The MLE-12 cells, a commonly used murine cell line expressing some type II alveolar epithelial cell markers, were either treated or not with coal dust (0 μg/mL, 200 μg/mL, 400 μg/mL, and 800 μg/mL) for 24 h. Microscopic fluorescent images were used to detect cell apoptosis or necrosis after annexin V/PI staining. Cells with the dual stain of annexin V/PI appear bright green in early apoptosis and red in late apoptosis, while the living and untreated (control) cells appear in a rare green color under a fluorescent microscope. **B** The counts of annexin V/PI positive cells (under a 400× magnification) at each dose are shown in the graph. ** C** Representative image of cleaved caspase 3 (CCP3) after immunofluorescent staining revealed apoptotic green cells after coal dust exposure. The untreated cells showed a dark field with negative CCP3 staining. DAPI was used as a counterstain for the nucleus. Apoptosis induced by coal dust in MLE-12 cells presented nuclear degeneration with condensed and damaged chromatin indicated by the DAPI stain. Coal dust was colocalized with cells under bright fields. The data values are expressed as Mean ± SEM. The data were derived from triplicate assays and two independent experiments. *p < 0.05 or **p < 0.05 compared with the untreated cells. **D** Heatmap of the apoptosis and necrosis expression of type-II alveolar epithelial cells in Gene Ontology (GO) terms. (**E**) GSEA plots of the top two differentially expressed regulons between coal dust (n = 963 cells) and PBS lungs (n = 1900 cells). The gene set enrichment analysis (GSEA) revealed that coal dust-exposed lungs highly enriched the ATP synthesis coupled proton of the collagen fibril organization and response to mechanical stimulus modules. The p-value was calculated using a permutation test (one-sided) based on phenotype, showing the statistical significance of the enrichment score

To verify the toxicity of coal dust in vitro, we used the mouse AT2 epithelial cell line MLE-12. MLE-12 cells grown on polylysine-coated slides in 24-well-plates were supplemented with a complete culture medium containing 10% FBS before they were exposed to 0 μg/mL, 200 μg/mL, 400 μg/mL, 800 μg/mL and 1000ug/ml coal dust for 24 h. With the increase of exposure concentration, the inhibition of MLE-12 gradually increased and finally reached causing 50% inhibition of cell survival (CC50) at 1000 μg/ml (Additional file 6: Fig. S6A). Then we studied the effect of coal dust exposure time on cytotoxicity. At the highest concentration of which the cells were not significantly inhibited, i.e. 200ug / ml, the cell inhibition rate did not change within 36 h (Additional file 6: Fig. S6B). Meanwhile, we analyzed cell apoptosis using an annexin V/PI apoptosis detection kit under confocal laser microscopy. FITC-conjugated annexin V and propidium iodide (PI) staining were used to discriminate between early apoptotic and late apoptotic/necrotic cells (Fig. 5A). The MLE-12 cells treated with 200 µg/mL had the highest number of green-colored apoptotic cells after 24 h post-exposure relative to the 400 μg/mL or 800 μg/mL groups, indicating that AT2 epithelial cells were sensitive to the stimulation of coal dust at a low dose. Conversely, cells treated with 400 μg/mL had the highest number of red-colored late apoptotic/necrotic cells, indicating that the limit of the lethal concentration of coal dust in AT2 cells was 400 μg/mL (Fig. 5B). Additionally, we verified AT2 apoptosis in vitro using the immunofluorescent staining of anti-caspase3 antibody (green fluorescence) and found that CCP3 positive AT2 cells in the coal dust group had obvious nuclear pyknosis and fragmentation (arrowhead) (Fig. 5C).

We believe that epithelial cells are the first line of defense (as opposed to immune cells), and any damage to them causes pro-inflammatory factor release, which initiates the body's immune response. The apoptosis/necrosis pathways were further analyzed following the in vitro experiment of epithelial cells. The proliferated AT2 cells were apoptotic with a higher signal of regulation of DNA fragments or necrosis relative to the control cells. Furthermore, newborn AT2 cells showed high sensitivity to the tumor necrosis factor response. (Fig. 5D). GSEA was performed on the DEGs of epithelial cells. Nine-month-old coal-exposed AT2 epithelial cells exhibited declined outside-related transcription levels after mechanical stimulation. Moreover, the mitochondrial transport, protein coupling, and intracellular ATP increased (Fig. 5E), suggesting that energy metabolism disorder is a critical link in the toxic process of coal dust that leads to cell death.

AT2 epithelial cells are a few essential cells, accounting for only 5% of the total lung cells, but they can proliferate and differentiate into AT1 epithelial cells, which repair alveolar structures. After the coal dust treatment, the alveolar type II cells proliferated significantly, indicating that their repair function was activated. However, our transcriptomes disclosed that proliferated AT2 showed functional heterogeneity, with reduced antibacterial activity, functional loss, and abnormal proliferation. Thus, the space-occupying lesions of coal dust in the lung interstitium may have caused abnormal processes or initiated their reprogramming. Nevertheless, the time-series analysis suggested that the newly generated AT2 epithelial cells were still highly active, which may be critical in inducing lung consolidation.

### Alveolar macrophages show rheostatic phenotypes and M2 polarization in mice with coal pneumoconiosis

**Fig. 6:**
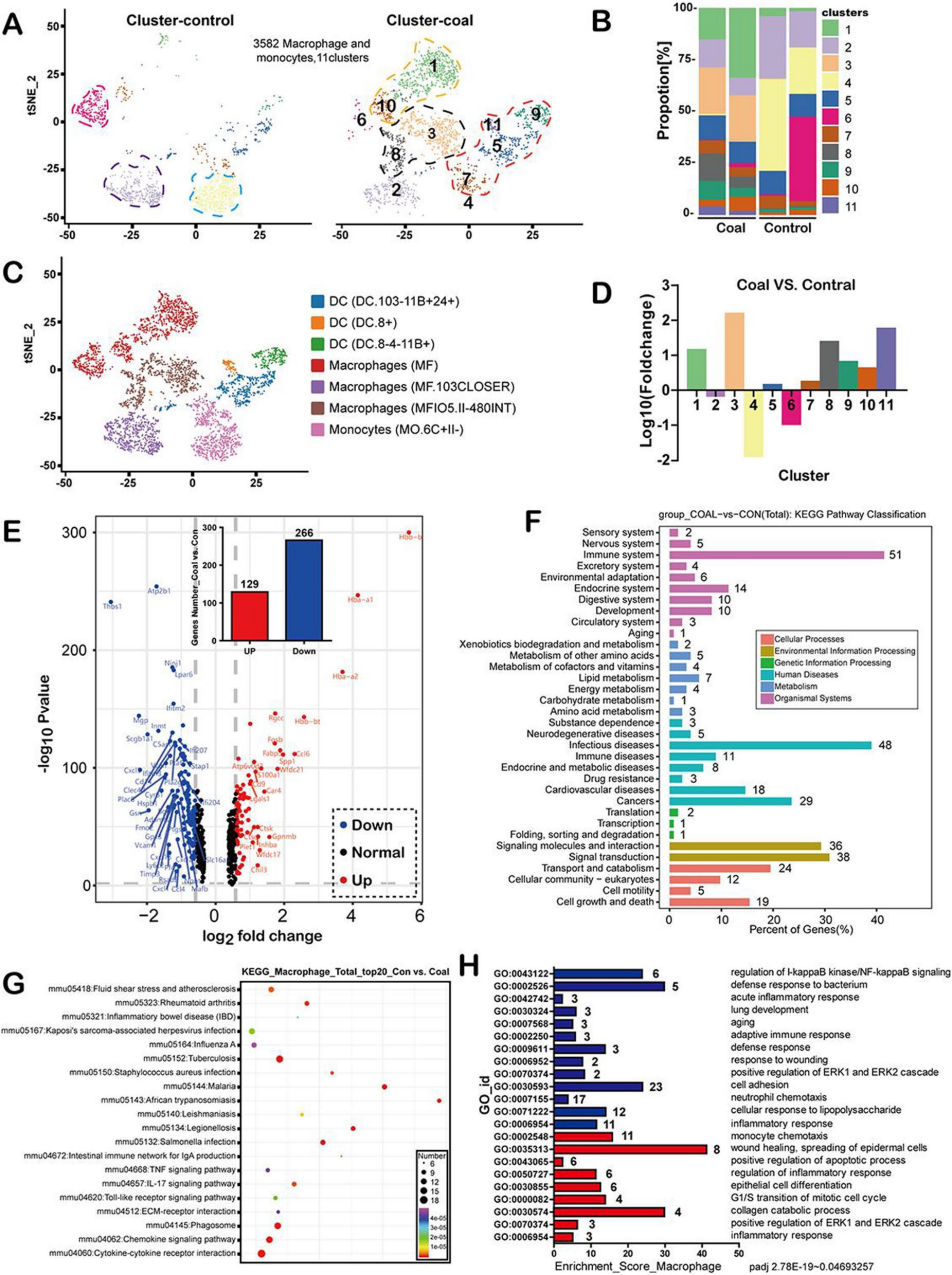
After long-term coal dust exposure, the heterogeneity, phenotypic diversity, and functional alternation of pulmonary macrophages. **A** tSNE plot view of 3582 macrophages, color-coded by the associated cluster (top) or the assigned cell type (bottom). **B** The fraction of macrophage subclusters from each of the four mice, box plots of the number of cells (with the plot center corresponding to the median). **C** The relative increase or decrease of the macrophages of clusters in the coal-exposed lungs was observed, compared with the control (positive represents more, negative represents less); **D** Single-Cell RNA-SEQ identified seven macrophage populations in mouse lungs. The tSNE diagram shows an aggregation of 3582 cells from 4 mice. **E** The volcano plot of the SDEGs of pulmonary macrophages from the coal-exposed mice and the control. A total of 129 upregulated genes are presented in red, while 266 downregulated genes are presented in blue (bar chart). **F** Kyoto Encyclopedia of Genes and Genomes **(**KEGG) pathway classification map. The genes were divided into six branches according to the biological pathways. **G** The top 20 significantly pathways are enriched by KEGG based on significantly differentially expressed genes (SDEGs). **H** The Gene Ontology (GO) enrichment analysis shows the association between the genes and phenotypes of coal dust-exposed mice

Mononuclear macrophages in the lung play a critical role in maintaining tissue homeostasis and controlling the inflammatory response to dust inhaled from the environment and lung disease pathogenesis [46]. To define the transcriptional landscape of lung-resident macrophages, we partitioned 3,582 transcriptionally distinct mononuclear macrophages into 11 clusters, which were shown in the coal dust and control groups (Fig. 6A). The cell-type composition of macrophages differed substantially in mice with coal pneumoconiosis (Fig. 6B, D). In all clusters, essential marker genes were highly expressed, while markers of other cell types were minimally expressed, except for OGN genes (Additional file 2: Fig. S2). A dissection of the heterogeneous origins of mouse alveolar macrophages revealed that these were macrophages, monocytes, and dendritic cells (Fig. 6A, bottom). The mixed profile of alveolar macrophages (AMs) represents phenotypic plasticity in response to inhaled coal dust. Here, two types of macrophages were detected in the normal mouse lung. It has been reported that AMs highly express the MARCO, FABP4, and MCEMP1 genes. The other type, interstitial macrophages, is derived from circulating monocytes. These cells are functionally different from tissue-resident macrophages, recruiting and expressing profibrotic genes during lung fibrosis [47]. Next, the AM type in lung tissues, including anti-inflammatory AM (C2 and 8, APOE^+^, CD163^+^, and C1qb^+^), pro-inflammatory AM (C10, IL1β^+^, and C6 IL6^+^), and actively cycling AM expressing anti-inflammatory markers were found. Moreover, as expected, we confirmed the decline of macrophage type 1 (M1)/M2 in mice with 9-month (advanced-stage) coal pneumoconiosis, indicating the activation of adaptive anti-inflammatory repair in response to long-term coal dust exposure (Fig. 6C, Additional file 2: Fig. S2). Consistently, the lungs of mice with coal pneumoconiosis were strongly enriched in Macs (anti- and pro-inflammatory macrophages in clusters 1, 3, and 8). In addition, both normal lungs and those infected with coal pneumoconiosis contained clusters of C1qb^+^ macrophages (C2).

To investigate the functional role of coal dust-induced changes at the transcription levels in macrophages, we identified 395 significantly differentially expressed genes (SDEGs), which included 266 downregulated and 129 upregulated genes in the coal group relative to the control group (Fig. 6E, Additional file 8: Table S4). We marked the top 20 upregulated and downregulated genes between the coal and control groups according to the log_2_FC in Additional file 8: Table S2. Significantly downregulated genes included ATP2b1, Thbs1, Ninj1, Lpar6, Lfitm, Scgb1a1, Mgp, CD14, and Cxcl3. To further study the complex behavior of CMP macrophages, a pathway enrichment analysis of DEGs was applied to understand better the biological function of a gene and its interaction with others. The differential genes in the macrophages were involved in 37 subclasses of metabolic pathways in six broad categories. Next, the data analysis by the metabolic pathway database KEGG was performed (Fig. 6F). A high number of genes were found among these signaling pathways, including the immune system (51 genes), infectious diseases (48 genes), signal transduction (38 genes), cancers (29 genes), and cardiovascular diseases (18 genes). Metabolic pathways are involved in signal transduction during environmental changes.

Meanwhile, there were four pathways associated with immune and disease resistance, including signaling molecules and interaction (36 genes), transport and catabolism (24 genes), cell growth and death (19 genes), and cellular community–eukaryotes (12 genes). These pathways are involved in all aspects of mammalian immunization. In short, the experimental KEGG annotation pathway enrichment analyses for macrophages based on lineage-specific genes identified by scRNA-Seq strongly highlighted immune-induced regulatory pathways in the pathogenesis of coal pneumoconiosis in mice. This study’s genes and pathways associated with the immune system, signal transduction, and disease processes were similar to those previously reported for particle-induced pneumoconiosis [48–50]. Thus, studies on immune-related genes and pathways identification here can advance our understanding of the molecular immune mechanisms in coal dust-activated CMP.

KEGG pathway enriched and analyzed all important signal transduction pathways and biochemical metabolic pathways regulated by DEGs [51]. 196 KEGG pathways annotated SDEGs of coal group/control group, and the first 20 enrichment pathways are shown in Fig. 6G (Additional file 8: Table S5 in details). We demonstrated six significantly enriched KEGG pathways, including the phagosome, tuberculosis, cytokine-cytokine receptor interaction, chemokine signaling pathway, IL-17 signaling pathway, and *Salmonella* infection pathways. The most significant enrichment signal is the cytokine—cytokine receptor interaction pathway, including 18 SDEGs such as interferon-gamma receptor 1 (Ifngr1), Ccl17, Ccl24, Ccl4, Ccl6, Ccl9, Ccr5, Cxcl1, Cxcl10, Cxcl13, Cxcl2, Cxcl3, Cxcr4, and more. In addition, we confirmed that autophagosome markers LAMP2, LC3B, and Beclin1, which are related to phagocytic pathways, were mostly co-located or adjacent to M2 macrophages that phagocytic coal dust particles (Additional file 3: Fig. S3). The single-cell analysis-based dissection uncovered macrophage phenotype changes linked to functional state transitions in the 9-month coal-exposed mouse lungs.

The transcriptome of macrophages reveals changes responsible for immune defense and tissue repair. To reveal some of the pathways that might contribute to coal pneumoconiosis formation in macrophages, GO term analysis was performed on the DEGs of the CMP transcriptome to compare the results to the control group. The top 22 most significantly upregulated and downregulated transcripts are listed in Fig. 6H. Pathway analysis revealed that the most highly upregulated biological processes involved wound healing, the spread of epidermal cells (GO0035313), the positive regulation of the apoptotic process (GO0043065), epithelial cell differentiation (GO0030855), and collagen catabolic processes (GO003057). The repressed biological processes included processing in the regulation of l-kappaB kinase/NF-kappaB signaling (GO0043122), defense response to bacteria (GO0002526), cell adhesion (GO0030593), and defense response (GO0009611).

### The epithelial crosstalk with macrophage in mice with coal pneumoconiosis is predicted using ligand-receptor interaction analysis

**Fig. 7:**
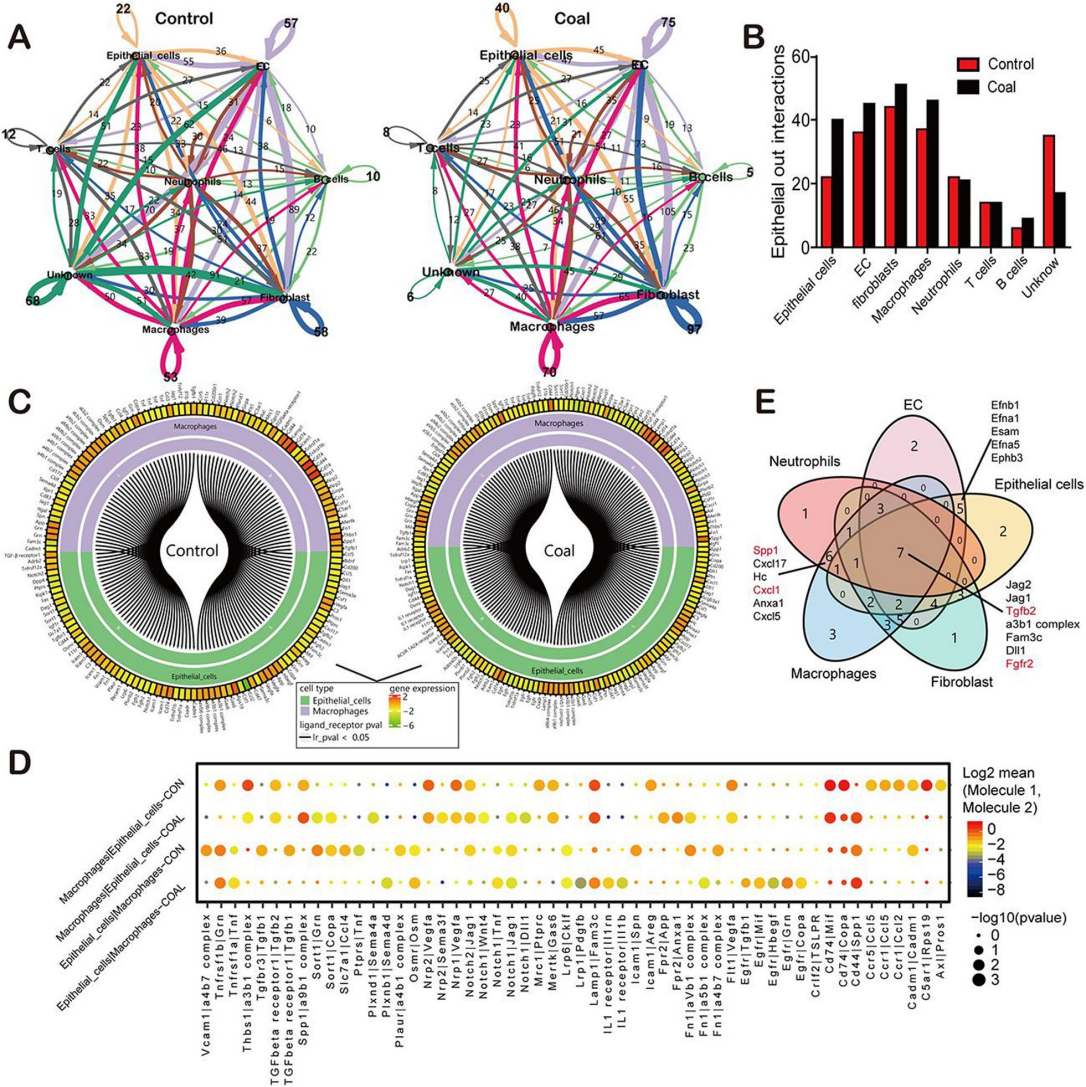
The crosstalk of Macrophage with Epithelial in response to long-term coal exposure. **A** The intercellular interactions among different cell types were shown in circos plot, which represents a ligand of one cell type directed to a corresponding receptor of another cell type. The thickness of each string corresponds to the number of receptor-ligand pairs, and color refers to cell type. **B** The numbers of significant ligand-receptor pairs between epithelial cells and other cells in coal dust-exposed (black bar) and control lung (red bar). **C** The computational method used to predict the macrophage-epithelial communication based on L-R interaction through scRNA-Seq provides prominent potential signaling in coal dust-exposed (right) and control (left) lungs. **D** Dot plot showing the mean level and percentage of selected interaction pairs associated with the response of epithelial cells and macrophages (directional). Each gene expression was considered independently for each sample source. **E** After the Single-cell transcriptome sequencing comparative analysis was performed, a five-set Venn diagram was used to depict unique and shared (overlapping circles) sets of differentially expressed genes (DEGs) in the coal-exposed lungs. Each ellipse shows the total number of coding sequences in one cell type. Intersections indicate predicted shared content

As we have successfully outlined the fate of epithelial cells and macrophages and delineated the molecular characteristics of different cell populations, we then infer a common ligand-receptor database mediated intercellular communication in advanced coal dust lung disease. CellPhoneDB was developed to predict the key signaling events between the spatially co-located cell groups by Efremova et al. [42]. The CellPhoneDB analysis performed on different epithelial cell types, including endothelial cells, fibroblasts, macrophages, neutrophils, and lymphocytes, showed that macrophages were the dominant communication hubs that secrete and reverse signals via 262 and 316 ligand-receptor pairs (Fig. 7A, E). After comparing ligand-receptor pairs with cell-specific genes, we classified possible ligand-receptor pairs in different cell populations (Additional file 8: Table S6-1, 6-2). Regarding ligand-receptor pairs (epithelium and macrophage specification) in the control and CMP groups, we found a stronger interaction relationship between the epithelial cells and macrophages in CMP (Fig. 7B, C).

To further predict the toxic damage mechanism in CMP lungs, we studied the signaling correspondence between epithelia and macrophages. After coal dust treatment, these cells co-located in space and actively signaled to each other. There were significant positive and negative signal states in macrophages, indicating that the cell state transition under coal dust exposure was highly regulated. Specifically, the TNF pathway showed rich signaling interactions among all four states, with CD74 receptors expressed primarily by macrophages. In addition, the dominantly ligand CD44 was contributing to dermal TGF signaling, which promotes granuloma formation by stimulating the in situ proliferation of mononuclear cells through autocrine and paracrine signaling [52]. Furthermore, macrophages secreted SPP1 to multiple cell surface receptors, consistent with the known role of physiological and pathological processes, including wound healing, inflammation, tumorigenesis, and ischemia. In addition, epithelial cells were the major ligand source for another important Notch signaling pathway that primarily expresses autocrine Notch1 and Notch2 in CMP.

The Cell Phone DB analysis also predicted that this dominant macrophage notch signaling was supplemented by a minor epithelial-derived Nothch2 ligand paracrine signaling, promoting macrophage M2 polarization, dependent on the interaction with CD47 and mediated by intracellular signaling through SHP-1 [53, 54]. Our analysis showed solid intercellular communication between macrophages and epithelial cells, including the innate immune system signaling members SIRPA and CD47, and Notch signaling ligands Jag1, gag2, Tgfb2, and Fam3c (Fig. 7D). These results further emphasize the indispensable roles of these well-defined pathways during coal dust lung specification. In addition, we observed robust ligand-receptor pairs within the epithelial cells and macrophage population, including SSP1, TGF-b, and CD74, indicating a robust autocrine relationship at this stage (Fig. 7C). However, some of these ligand-receptor pairs differed between epithelial cells and macrophages, suggesting heterogeneity in coal dust lung cells, as illustrated in our DEG analysis.

### The infiltrated M2-type alveolar macrophages with CD206^+^ and MLPH^+^ subset after the long-term coal exposure were significantly decreased after Vitamin D treatment

**Fig. 8:**
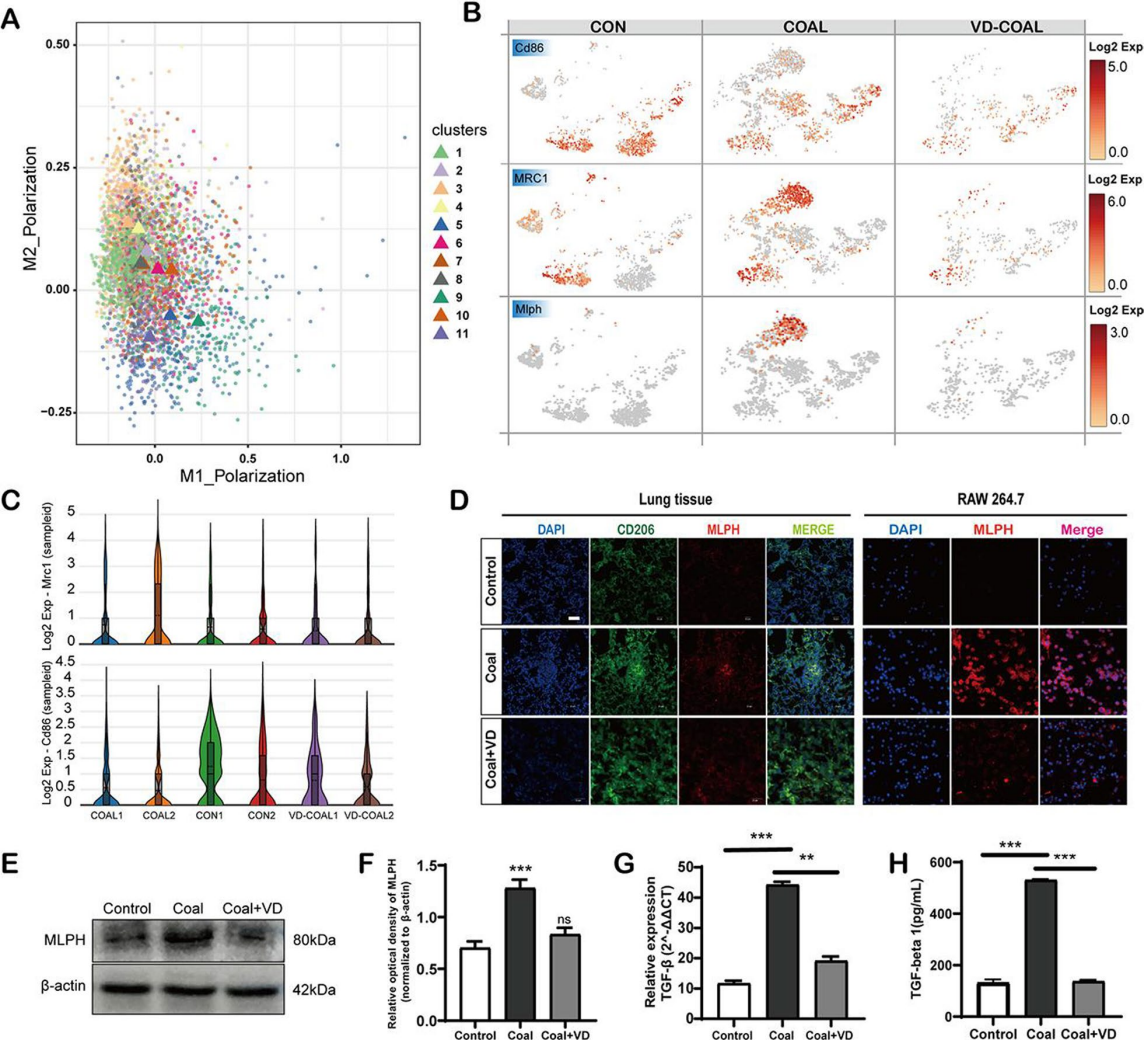
A distinctive M2 type activation in alveolar macrophages after coal exposure **A** A single-cell sequencing plot shows the effects of coal exposure and Vitamin D supplemented coal exposure on the polarization of macrophages to the M2-type. **B** The group’s distribution of canonical cell markers CD86, Mrc1, and MLPH target gene transcripts across the t-distributed stochastic neighbor embedding (tSNE) plot. The subset of M1 cells was identified by CD86 expression, and the M2 subset was identified by Mrc1 expression. The color key indicates MAGIC-imputed gene expression values. **C** Violin plots display the distribution of the expression of previously reported M2 polarization-related markers (Mrc1, up), M1 polarization-related markers CD86, bottom). **D** Double immunofluorescence staining of MLPH and CD206 verified M2-type macrophages that emerged after coal exposure and disappeared after the Vitamin D treatment in the lung tissue and Raw267.4 cells both in vivo and in vitro. Scale bar, 40 μm. **E** The RAW264.7 cells were treated with phosphate-buffered saline (PBS; control), coal dust, and a combination of coal dust and Vitamin D; the whole-cell lysates were harvested 24 h after the treatment. Western blots determined the expression level of MLPH. β-actin was used as the internal control

Next, we focused on markers of specialized biological features of macrophages. Using scRNA-Seq, the marker gene expression of macrophage clusters was shown in Additional file 4: Fig. S4 and Additional file 8: Table S7. The specialized M2 phenotypes expressing MLPH markers were found, further illustrating the specific M2 subset (Fig. 8B). Finally, alveolar macrophages in CMP lungs were polarized into the M2-type subset (Fig. 8A), and a fraction of this population was identified with double CD206^+^MLPH^+^ expression in vivo and in vitro. In addition, the scRNA-Seq showed that 9-month coal-dust exposed lung tissues had increased M2 cells and relatively decreased M1 cells compared to control groups; however, this trend was reversed in the VD_3_-treated group (Fig. 8C).

Here, we focused on CD86, Mrc1, and MLPH for two reasons. First, CD68 and Mrc1 represent two different identities of the classical macrophage classification of M1 and M2 [55]. Second, scRNA-Seq data suggested that the MLPH expression was explicitly increased in alveolar macrophages at 9-month CMP, but only increased in M2 cells. Using selective marker genes (CD86 for M1 and Mrc1 for M2), we were able to confirm the predicted emergence of MLPH^+^ macrophages and upregulated in M2 cells only in CWP lungs (Fig. 8B). Vitamin D exerts its beneficial effects on many macrophage components, modulating phagocytic activity and cytokine production [56]. To validate that VD_3_ accurately regulated gene expression in specific macrophage populations, we performed in situ immunofluorescent staining (Fig. 8D) and western blot (Fig. 8E, F) of the samples from the same tissues used for single-cell RNA-Seq analysis. The result showed that the MLPH expressed in CMP lungs increased significantly more than in the control lungs but markedly decreased after the Vitamin D treatment. Furthermore, the qPCR and ELISA analysis revealed that coal-exposed macrophages had increased TGF-β expression and secretion but decreased after the VD_3_ treatment (Fig. 8G, H). TGF-β plays a critical role in lung inflammation and is a recognized indicator of fibrosis. Our results suggest that VD_3_ may be a potential alternative treatment for alleviating pulmonary fibrosis caused by coal dust. These results also support the feasibility of the supplement of the VD_3_ in patients with coal pneumoconiosis.

## Discussions

Coal dust-induced alveolar structural damage and repair are major challenges in lung disease. Although it has been reported that AT2 epithelial cells have significant regeneration capacity, this regeneration process leads to abnormal or stunted repair [12, 57]. Herein, we provide new insights into the single-cell analysis. A new population of HAAS emerged in the long-term coal-exposed mouse lungs, which lost antibacterial pathways, including CHI3L1, APOC1, CXCL15, IL33, and LYZ1, and played an important role in pulmonary immunity. IL-33 is a significant driver of cellular differentiation and lung maturation, produced mainly in AT2 cells [58], which binds uniquely to IL1rl1 in adjacent basophils of lung tissue. The proportion of AT2 epithelial cells was small in normal lungs, but there was an abnormal increase after coal dust exposure. Furthermore, newborn AT2 cells produce more disease-associated factors, such as IL33. IL-33 specifically primes basophils and mast cells and affects AM maturation toward M2-type polarization [59, 60]. IL33 is also a marker of repair promotion and is expressed in AT2 during lung development and maturation [61]. It is also a significant factor that regulates hypoxia-induced lung injury and can mediate hypoxia-inducible factors [62]. Fate-mapping studies indicate a clear lineage relationship between AT2 and HAAS. Therefore, we amended the hierarchy of alveolar epithelial cells involving these convertible cell states. Specific gene expression signatures demonstrate that HAAS gradually loses AT2 characteristics and represents a unique convertible population. The HAAS of the human lung has been previously identified and is especially apparent in the fibrotic regions of IPF lungs. Regarding human HAAS cells, HAAS-like cells in the present murine lungs were characterized by enrichment genes related to cellular senescence, Notch signaling, and WNT-regulated genes, all of which are linked to lung fibrosis [25, 63]. In contrast, we found a high expression of TRP63, KRT5, and COL1A1 in the epithelial cells of mice with coal pneumoconiosis; these were previously found only in human HAAS cells.

We found that AT2 cells undergo broad stimulation and deformation after coal dust entering the alveoli, causing them ready for DNA damage, a feature of most lung diseases with pulmonary fibrosis [12]. Previous studies have revealed that respirable particles lead to DNA damage once they move or remain in the pulmonary interstitium [64–66]. Our in vivo and in vitro study suggests that mouse AT2 cells undergo extensive stimulation and deformation during exposure to coal dust particles. Therefore, this specific AT2 cell state induced by the coal dust implies DNA damage in lung diseases.

The lungs efficiently match air and blood within the vast area of the alveolar surface, ensuring oxygen intake and delivery. Coal dust induced-hypoxia is a crucial driver of Notch signaling activation in epithelial stem/progenitor cells. It has previously been reported that hypoxia can induce Notch activity in vitro [67]. Our single-cell sequencing analysis showed that coal pneumoconiosis activates Notch activity. Using CellphoneDB, we found that anoxic/notch-activated signals in the epithelium and macrophages communicate with a group of genes controlling motility and invasion. Nevertheless, taken together, these data paint a pneumoconiosis epithelium and macrophage picture suggestive of more preventative, coactive, and reparative roles on their part, crucial in maintaining lung function and homeostasis. Future studies are required to characterize the potential of alveolar progenitors to drive a regenerative alveolar process and decode the immune microenvironment that favors AT2 proliferation.

A combination of single-cell transcriptome sequencing and immunofluorescence studies confirmed that disease-relevant MLPH^+^ macrophages are connected by a branched lineage that proceeds from coal dust-induced M2 polarization. We found that the homeostatic alveolar macrophages in mice rarely contained the MLPH^+^ subset but expressed highly CD86. However, coal exposed-alveolar macrophages had a large proportion of the CD206^+^ MLPH^+^ double staining subset, which may ultimately lead to pulmonary fibrosis. Interestingly, MLPH is involved in intracellular substance transport and becomes an upstream complex of TGF-β signaling. In addition, we deconstructed the VD3 supplement by scRNA-Seq as a comprehensive model and analyzed the effects of VD3 on macrophages to the subset level. Our scRNA-Seq data suggest that the coal-induced macrophage M2 polarization is suppressed by VD_3_ supplementation, which defines VD_3_ as a key to homeostatic M1/M2 of murine macrophages in a manner that involves other activities but does not exclude anti-fibrosis. This important function of VD_3_ in macrophage therapy may benefit from their hormone activity alone or their ability to coordinate both paracellular and transcellular transport activity across the other cells.

Taken together, we uncovered a highly active AT2 state (HAAS), an abnormal increase that spans between AT2 and AT1. Notable, we further identified coal pneumoconiosis macrophage niches and clarified multiple functional enrichment pathways for alveolar macrophages, through which activated alveolar macrophages facilitate two-way communication with epithelial cells. Our results indicated M2-type macrophage polarization in long-term coal-exposed mouse lungs and disclosed an M2-macrophage subset with MLPH^+^CD206^+^ expression that significantly decreased after the administration of the Vitamin D_3_ treatment. These findings provide potential alternative targets for CWP in the future. This study was based on a mouse model of coal pneumoconiosis. Next, we will validate the molecular mechanism discovered by single cell sequencing in human pneumoconiosis samples.

## Conclusions

This study successfully established a reliable coal pneumoconiosis mouse model to study the cytotoxicity of respiratory coal dust particles to lung epithelial cells and macrophages in coal mines. After 9 months of coal dust stimulation, pulmonary inflammation and pulmonary fibrosis were significantly induced. Single-cell transcriptome sequencing technology allows the characterization of whole lung cells and molecules and provides a more comprehensive picture of coal-dust-induced lung cell changes and possible signal communication than traditional mechanism studies. Our study is the first to show that chronic coal dust stimulation can lead to substantial heterogeneity of epithelial cells and macrophages, and induce cellular inflammatory and death pathways, thereby promoting the progression of pulmonary fibrosis. Among them, HAAS, the highly active state of epithelial cells with no antibacterial ability, suggested the repair imbalance of lung epithelial cells. In addition, a subset of M2-type macrophages generated by the polarization of M2 macrophages induced by coal dust could be inhibited by VD, which may be related to the alleviation of the pulmonary fibrosis process by VD.

## Supplementary Information

**Additional file 1**. **Figure S1**. Macrophage annotation and identification. (A) Visualized transcriptome cluster by tSNE. (B) Expression levels of the indicated genes projected onto tSNE in Clasters10,11,17. (C) Overview of violin plot for expression of critical lineage-associated genes by macrophages (number of cells in each cluster displayed in B).

**Additional file 2**.** Figure S2**. Distribution of two subsets of functional macrophages types across tSNE plot. Macrophages expressed pro-inflammatory (CD207^+^, IL1β^+^, TNF^+^, IL6^+^) and anti-inflammatory (IL10^+^, APOE^+^, CD163^+^, C1qb^+^) genes.

**Additional file 3**. **Figure S3**. IHC analysis of CD206, Beclin1, LC3B, LAMP2 in coal dust-exposed lung. Data values represent (Mean ± SEM) obtained from two independent experiments in triplicate assays. The graph showed the quantification of intensity when comparing the sections from the coal group with the control ones (*p < 0.05, *p < 0.01, *p < 0.001): scale bar, 50 μm.

**Additional file 4**. **Figure S4**. The heat map showed the top 10 markers of macrophage.

**Additional file 5**. **Figure S5**. Pulmonary score and Lung function test of mice exposed to coal dust for nine months. (A) Pathology score performed by Roderick J. Pathology classification standard of lung injury at three, six, nine-months. (B) Pulmonary fibrosis score performed by Ashcroft Criteria for grading lung fibrosis at three, six, nine -months. (C) Body weight recorded throughout the nine months. (D) The main indexes of lung function are Penh: enhanced pause; Mv: minute volume; Av: accumulated volume; EF50: expiratory flow 50%; PIF: peak inspiratory flow; PEF: peak expiratory flow; F: frequency; Rpef: ratio of time to peak expiratory flow (*p < 0.05, **p < 0.01, ***p < 0.001).

**Additional file 6**. **Figure S6**. Toxic effects of coal dust on MLE-12 cells. Causing 50% inhibition of cell survival (CC50) of coal dust was measured in MLE-12 cells treated with different dose (A) and different time point at 200 μg/ml (B) (*P < 0.05, **P < 0.01, ***P < 0.001, Treatment vs Control).

**Additional file 7**. Advanced analytical methods.

**Additional file 8**. Raw datasets.

## Data Availability

All data related to this study are publicly available upon reasonable request to the corresponding author.

## Abbreviations

AMs: Alveolar macrophages
AP: Alkaline phosphatase
AT1: Alveolar type 1 cells
AT2: Alveolar type 2 cells
BMDMs: Bone marrow-derived macrophages
CCP3: Cleaved caspase 3
CMP: Coal pneumoconiosis in mice
CWP: Coal worker’s pneumoconiosis
DEGs: Differentially expressed genes
GEMs: Gel beads in emulsions
GO: Gene Ontology
GSEA: Gene set enrichment analysis
GSVA: Gene set variation analysis
HAAS: Highly active AT2 state
HE: Hematoxylin and eosin
IHC: Immunohistochemistry
PBST: Phosphate buffered saline with Tween
BSA: Bovine serum albumin
ATP: Adenosine triphosphate
AAT2: Actived alveolar epithelial cell type 2
CDPs: Coal dust particles
FN: Fibronectin
RFs: Repair related factor
BEC: Blood endothelial cells
ExCM: Extracellular matrix
IM: Interstitial macrophages
KEGG: Kyoto Encyclopedia of Genes and Genomes
KRT5: Keratin 5
M1: Macrophage type 1
M2: Macrophage type 2
MSHA: Mine Safety and Health Administration
NS: Normal saline
PBS: Phosphate-buffered saline
PCA: Principal component analysis
PI: Propidium iodide
PMF: Progressive massive fibrosis
PVDF: Polyvinylidene difluoride
SCRNA-SEQ: Single-cell RNA sequencing
scRNAseq: Single-cell transcriptomics
SDEGs: Significantly DEGs
SPC: Surfactant protein C
tSNE: T-distributed stochastic neighbor embedding
qPCR: Quantitative real-time PCR
UMI: Unique molecular identifier
