# Supplementary figures and images for: Coal dust exposure triggers heterogeneity of transcriptional profiles in mouse pneumoconiosis and Vitamin D remedies
Source: Part Fibre Toxicol. 2022 Jan 20;19:7. doi: 10.1186/s12989-022-00449-y (PMC8772169; doi:10.1186/s12989-022-00449-y)

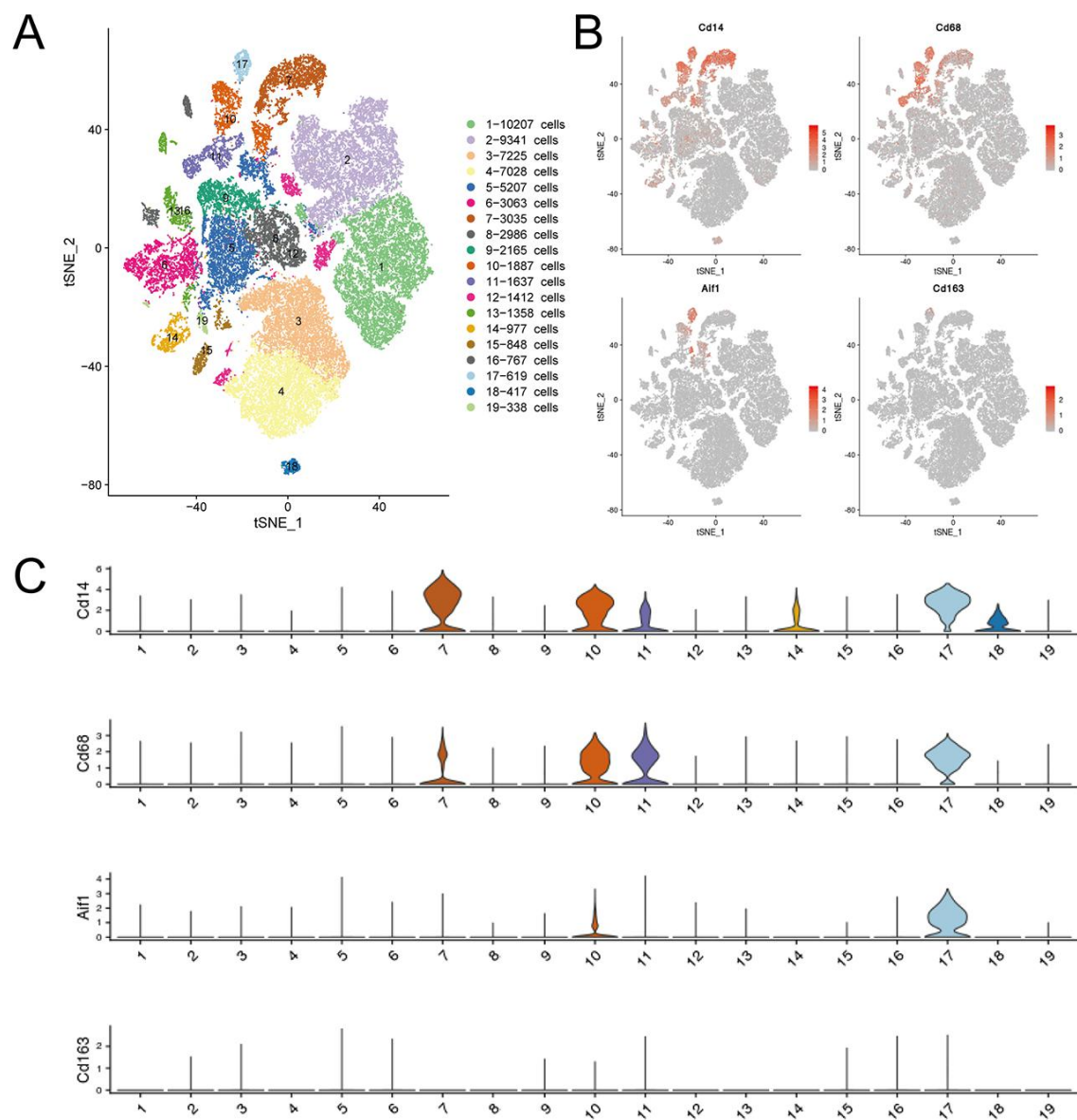

Supplement: Supplementary file 1 — Additional file 1. Figure S1. Macrophage annotation and identification. (A) Visualized transcriptome cluster by tSNE. (B) Expression levels of the indicated genes projected onto tSNE in Clasters10,11,17. (C) Overview of violin plot for expression of critical lineage-associated genes by macrophages (number of cells in each cluster displayed in B). [file 12989_2022_449_MOESM1_ESM.pdf]

Alveolar macrophages (AM)

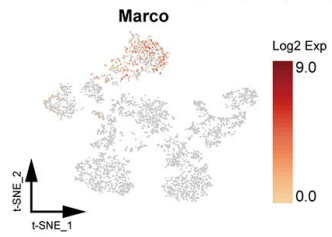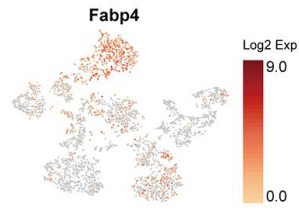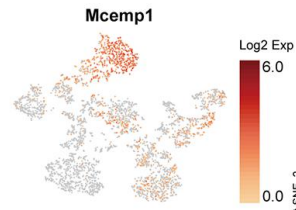

Ogn+ macrophages

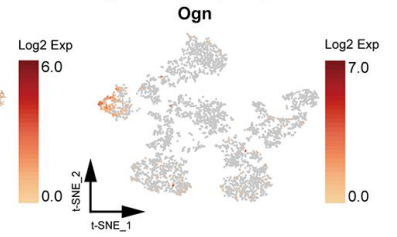

pro-inflammatory

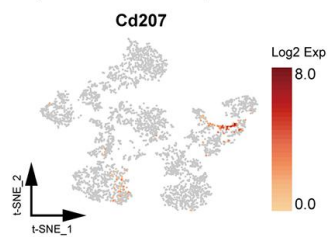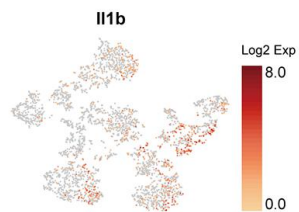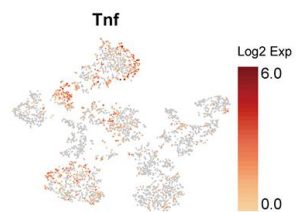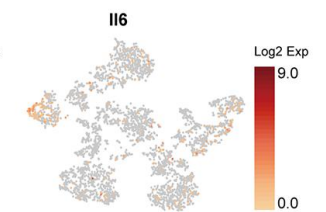

anti-inflammatory

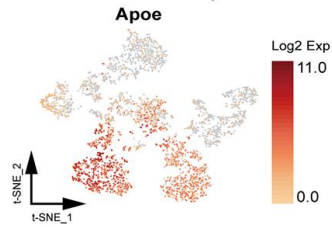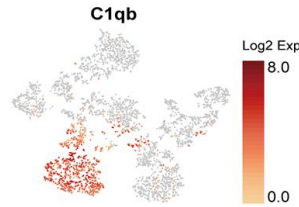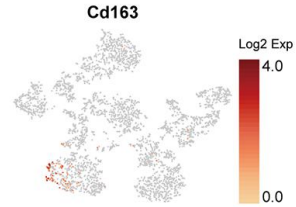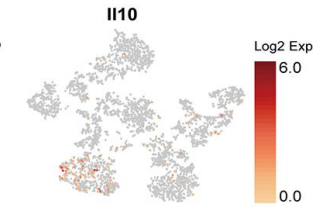

Supplement: Supplementary file 2 — Additional file 2. Figure S2. Distribution of two subsets of functional macrophages types across tSNE plot. Macrophages expressed pro-inflammatory (CD207+, IL1β+, TNF+, IL6+) and anti-inflammatory (IL10+, APOE+, CD163+, C1qb+) genes. [file 12989_2022_449_MOESM2_ESM.pdf]

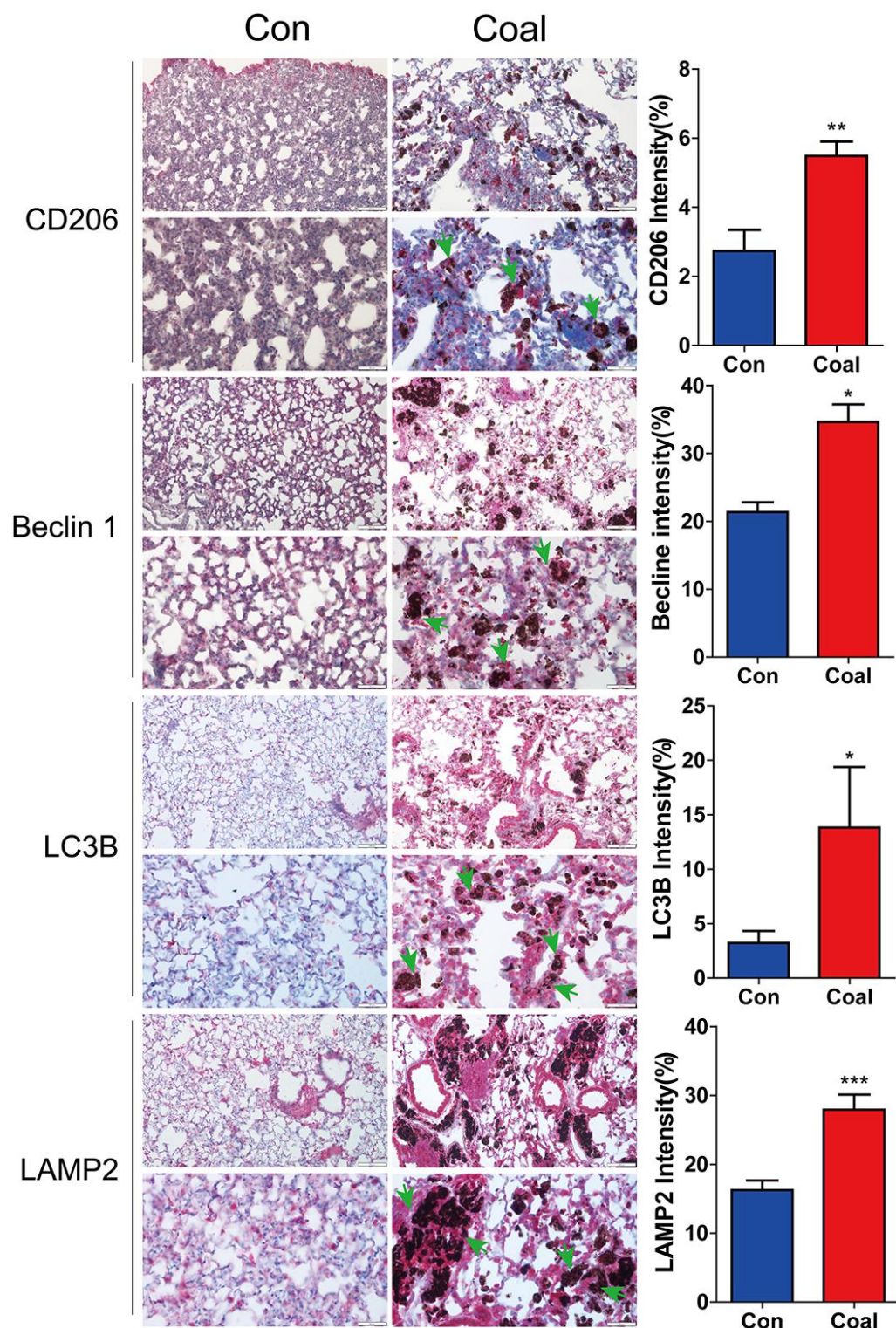

Supplement: Supplementary file 3 — Additional file 3. Figure S3. IHC analysis of CD206, Beclin1, LC3B, LAMP2 in coal dust-exposed lung. Data values represent (Mean ± SEM) obtained from two independent experiments in triplicate assays. The graph showed the quantification of intensity when comparing the sections from the coal group with the control ones (*p < 0.05, *p < 0.01, *p < 0.001): scale bar, 50 μm. [file 12989_2022_449_MOESM3_ESM.pdf]

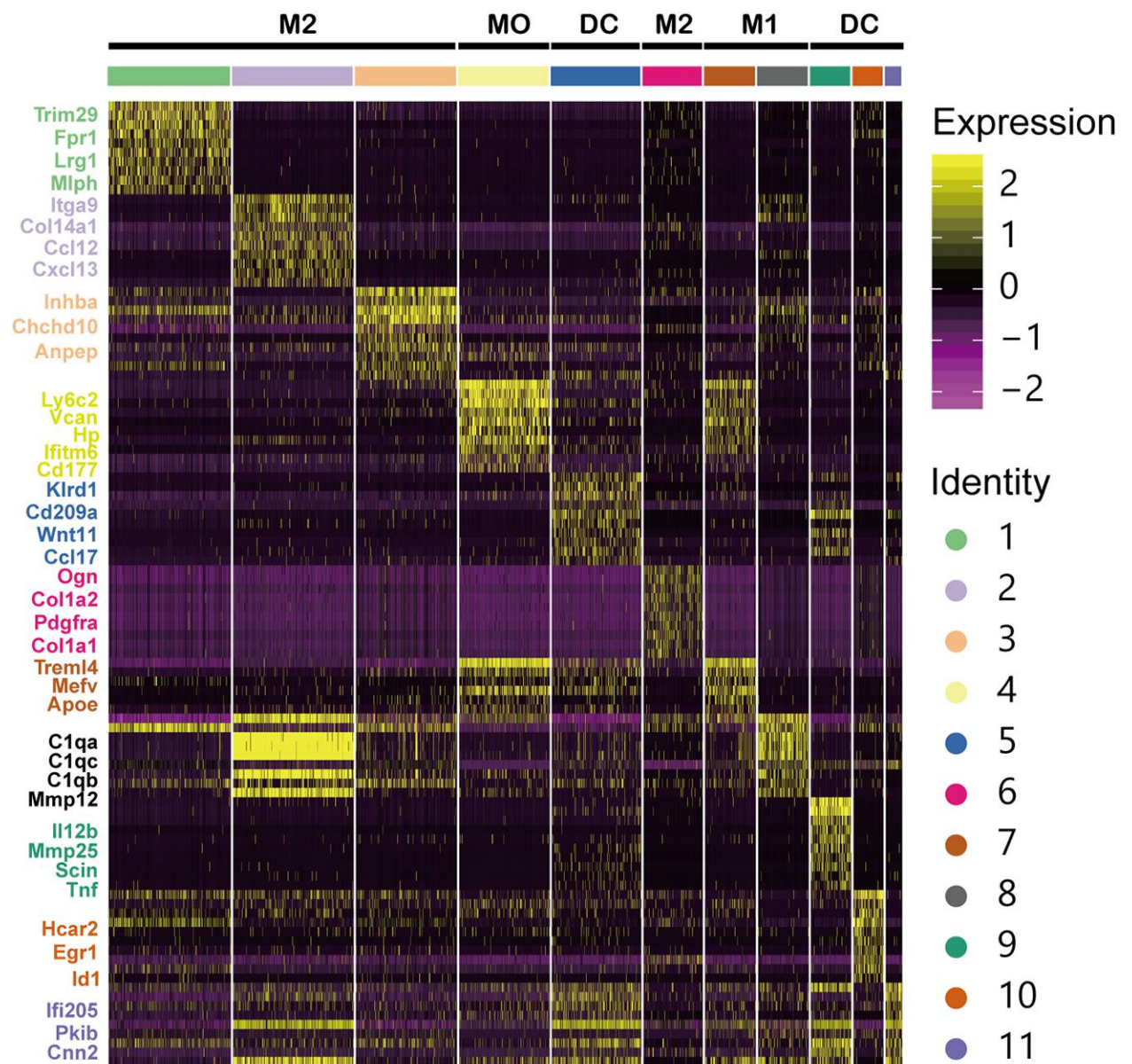

Supplement: Supplementary file 4 — Additional file 4. Figure S4. The heat map showed the top 10 markers of macrophage. [file 12989_2022_449_MOESM4_ESM.pdf]

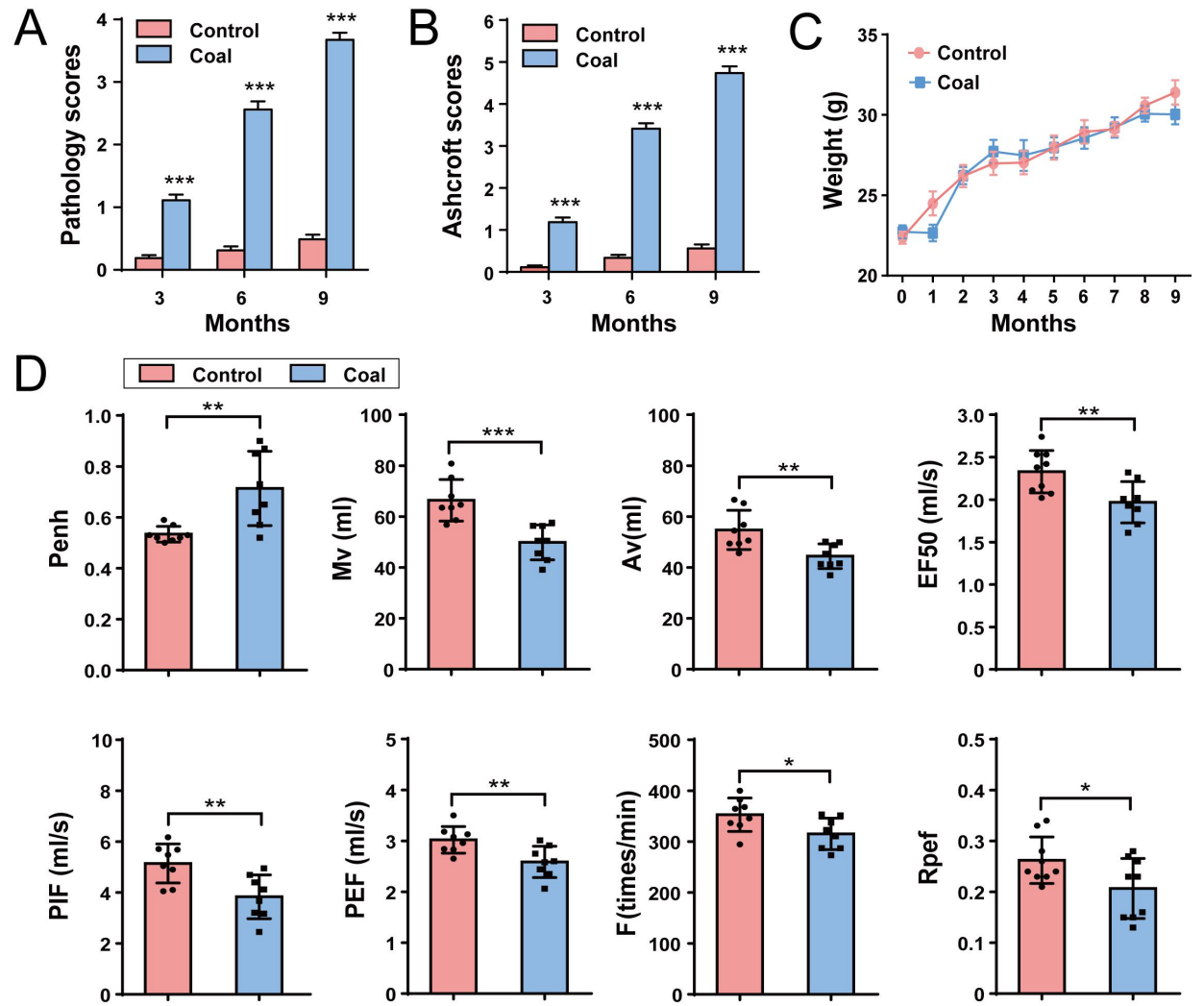

Supplement: Supplementary file 5 — Additional file 5. Figure S5. Pulmonary score and Lung function test of mice exposed to coal dust for nine months. (A) Pathology score performed by Roderick J. Pathology classification standard of lung injury at three, six, nine-months. (B) Pulmonary fibrosis score performed by Ashcroft Criteria for grading lung fibrosis at three, six, nine -months. (C) Body weight recorded throughout the nine months. (D) The main indexes of lung function are Penh: enhanced pause; Mv: minute volume; Av: accumulated volume; EF50: expiratory flow 50%; PIF: peak inspiratory flow; PEF: peak expiratory flow; F: frequency; Rpef: ratio of time to peak expiratory flow (*p < 0.05, **p < 0.01, ***p < 0.001). [file 12989_2022_449_MOESM5_ESM.pdf]

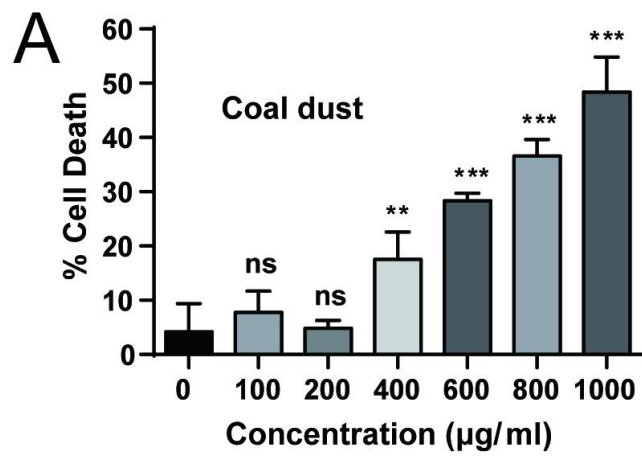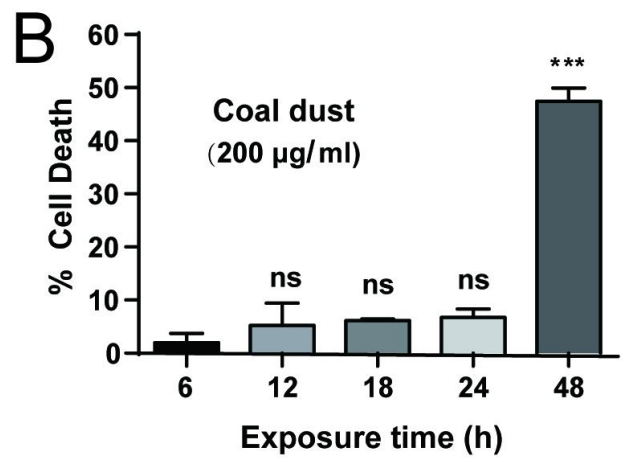

Supplement: Supplementary file 6 — Additional file 6. Figure S6. Toxic effects of coal dust on MLE-12 cells. Causing 50% inhibition of cell survival (CC50) of coal dust was measured in MLE-12 cells treated with different dose (A) and different time point at 200 μg/ml (B) (*P < 0.05, **P < 0.01, ***P < 0.001, Treatment vs Control). [file 12989_2022_449_MOESM6_ESM.pdf]
