# Supplementary material for: Coal dust exposure triggers heterogeneity of transcriptional profiles in mouse pneumoconiosis and Vitamin D remedies
Source: Part Fibre Toxicol. 2022 Jan 20;19:7. doi: 10.1186/s12989-022-00449-y (PMC8772169; doi:10.1186/s12989-022-00449-y)
Supplement: Supplementary file 7 — Additional file 7. Advanced analytical methods. [file 12989_2022_449_MOESM7_ESM.docx]

**2.10.1 Tissue processing**

C57BL/6 mouse lung tissues were resected, matched coal spot and adjacent normal samples were collected from each individual and transported immediately to the research facility. All samples were obtained with informed consent and approval from the Animal Ethics Committee of the Anhui University of Science and Technology. Each sample was subsequently minced on ice to less than 1-mm cubic pieces, followed by enzymatic digestion using DNase I (Worthington; 30 U/ ul), collagenase IV (Worthington; 195 U/ul), collagenase I (Worthington; 10 U/ul), and 30% FBS for 1 h at 37 °C. During the time, samples were manually shaken every 5 min. Next, samples were centrifuged at 300 rcf for 30 s at room temperature, and supernatants were removed without disturbing the cell pellet. 1× PBS (calcium and magnesium free) containing 0.04% weight/volume BSA (400 µg/mL) was then added and the mixture was centrifuged at 300 rcf for 5 min. The resulting cell pellet was resuspended in 1 mL of red blood cell lysis buffer and incubated for 10 min at 4 °C. After that, samples were resuspended in 1 mL PBS containing 0.04% BSA and filtered by using Scienceware Flowmi 40-µm cell strainers (VWR). Finally, cell concentration and cell viability were determined by hemocytometer and Trypan Blue staining.**2.10.2 Single-cell RNA sequencing** Single-cell RNA-seq libraries were prepared with Chromium Single Cell 3’ Reagent v3 Kits according to the manufacturer’s protocol. Single-cell suspensions obtained in the previous step were loaded on a Chromium Single Cell Controller Instrument (10× Genomics), where single cells were suspended in calcium- and magnesium-free PBS containing 0.04% weight/volume BSA to prepare gel bead in emulsions (GEMs). Within each GEM, barcoded full-length cDNAs were synthesized by reverse transcription reactions, followed by the disruption of emulsions using the recovery agent. cDNAs were then amplified by PCR with appropriate cycles, depending on the recovery cells. Finally, amplified cDNA was fragmented, end-repaired, A-tailed, index adaptor ligated, and library amplified for next-gen sequencing by the MGI200 platform with 100-bp paired-end reads.**2.10.3 Single-cell RNA-seq data preprocessing**Cell Ranger software suite (version 3.0.0, 10x Genomics) was used to process the raw data, including demultiplexing using cellular barcodes, read mapping, and down-sampling across samples for data aggregation. The resulting unique molecular identifier (UMI) count matrix was next fed into the R package Seurat (version 3.1.0) for downstream analyses. Multiplets were filtered using a heuristic approach, where cells with an average UMI count per gene two standard deviations away for the population mean were assigned as possible multiplets and removed. Cells with >20% of counts from mitochondrial genes were also filtered out, resulting in 50,767 single cells with high quality data. Count matrix was further normalized by library size for downstream analyses. Next, top variable genes across single cells were selected using a previously described method [Macosko et al] and their normalized counts was log transformed, followed by dimensionality reduction using MNN. Cell clusters were then generated by a graph-based approach and visualized in two dimensions derived from t-distributed stochastic neighbor embedding (tSNE) [ref]. The likelihood ratio test that simultaneously tests for changes in mean expression and in the percentage of expressed cells was applied to identify significantly differentially expressed genes among clusters.

**2.11 Advanced analysis methods**

**2.11.1 Analysis of sub-clusters in epithelial cells and macrophages** Following primary cell type annotation, epithelial cells, stromal cells, and immune cells were extracted via the “*SubsetData*” function in the Seurat package. For each cell type of interest, subclusters were further identified using the “*FindClusters*” function. To annotate those sub-clusters, cell markers with dominant expression were identified by the “*FindAllMarkers*” function with adjusted P value <0.01 and fold change >0.5 as described in previous studies.

**2.11.2 Functional enrichment analyses** Gene Ontology (GO) and Kyoto Encyclopedia of Genes and Genomes (KEGG) pathway analyses were performed using Metascape (http: //metascape.org)([Zhou et al., 2019](#_ENREF_60)). Notably, P < 0.01 and the number of enriched genes > 3 were adopted as the thresholds for identify pathway enrichment.**2.11.3** **Gene set variation analysis (****GSVA)The “***GSVA*” ([Hanzelmann et al., 2013](#_ENREF_16)) function implemented in the GSVA package (version 1.30.0) was used to assign pathway activities for each cell, with the gene set files preload using the GSEABase package (version 1.44.0) The differences in pathway activities among cell were compared using the LIMMA package (version 3.38.3).**2.11.4 Gene set enrichment analysis (GSEA)**

GSEA was performed on a list of genes ranked according to limma PI-values [-log10(adjusted p-value) × absolute logFC], using the GSEA *PreRanked* function with enrichment statistics and 1,000 permutations ([Reimand et al., 2019](#_ENREF_42)). A custom gene set for those genes was first created and merged with the GO BP category of the MSigDB database to obtain the enrichment ranking. Gene enrichment within a cluster was visualized by heatmap using Z-score of averaged expression per gene per cluster.

**2.11.5 Cell cycle analysis**The prediction of the cell cycle phase of individual cells was performed using the “*CellCycleScoring*” function in Seurat([Butler et al., 2018](#_ENREF_4)) . Briefly, cells expressing either the G2/M or the S phase gene markers were assigned into respective phases.**2.11.6 Pseudotime analysis**The developmental pseudotime was determined using the Monocle2 package([Trapnell et al., 2014](#_ENREF_49)). A Seurat object was converted to a CellDataSet object using the “*importCDS*” function in Monocle2, and genes informative in cell ordering along the pseudotime trajectory were selected by the *differentialGeneTest* function (qval < 0.01). Dimension reduction and clustering analysis were performed with the *reduceDimension* function, followed by trajectory inference using the *orderCells* function with default parameters. Gene expression was plotted by the *plot_genes_in_pseudotime* function to track changes over pseudo-time. **2.11.7 Cell-cell communication analysis** CellPhoneDB (v2.0) was used to identify biologically relevant ligand-receptor interactions from single-cell transcriptomic (scRNAseq) data ([Efremova et al., 2020](#_ENREF_13)). A ligand or a receptor was defined as “expressed” in a particular cell type if 10% of cells had non-zero read counts for the ligand/receptor encoding gene. To derive the statistical significance, cluster labels of all cells were randomly shuffled and the LR interaction score between two cell types was calculated to generate the null distribution for each LR pair. P-values were based on 1,000 permutations to define the cell-cell communication network, two cell types were linked if the ligand was expressed in onecell type and the receptor in the other. R packages Igraph and Circlize were used to display the cell-cell communication networks.
